# Exosome-based targeted delivery of NF-κB ameliorates age-related neuroinflammation in the aged mouse brain

**DOI:** 10.1038/s12276-024-01388-8

**Published:** 2025-01-20

**Authors:** Chae-Jeong Lee, Seung Hyun Jang, Jiwoo Lim, Hyunju Park, So-Hee Ahn, Seon Young Park, Hyangmi Seo, Soo-Jin Song, Jung-A Shin, Chulhee Choi, Heon Yung Gee, Youn-Hee Choi

**Affiliations:** 1 Department of Physiology, Inflammation-Cancer Microenvironment Research Center, Ewha Womans University College of Medicine, Seoul, 07804 Republic of Korea; 2 Department of Pharmacology, Brain Korea 21 PLUS Project for Medical Sciences, Yonsei University College of Medicine, Seoul, 03722 Republic of Korea; 3 ILIAS Biologics Inc., Daejeon, 34014 Republic of Korea; 4 Department of Anatomy, Ewha Womans University College of Medicine, Seoul, 07804 Republic of Korea

**Keywords:** Ageing, Chronic inflammation

## Abstract

Neuroinflammation, a significant contributor to various neurodegenerative diseases, is strongly associated with the aging process; however, to date, no efficacious treatments for neuroinflammation have been developed. In aged mouse brains, the number of infiltrating immune cells increases, and the key transcription factor associated with increased chemokine levels is nuclear factor kappa B (NF-κB). Exosomes are potent therapeutics or drug delivery vehicles for various materials, including proteins and regulatory genes, to target cells. In the present study, we evaluated the therapeutic efficacy of exosomes loaded with a nondegradable form of IκB (Exo-srIκB), which inhibits the nuclear translocation of NF-κB to suppress age-related neuroinflammation. Single-cell RNA sequencing revealed that these anti-inflammatory exosomes targeted macrophages and microglia, reducing the expression of inflammation-related genes. Treatment with Exo-srIκB also suppressed the interactions between macrophages/microglia and T and B cells in the aged brain. We demonstrated that Exo-srIκB successfully alleviates neuroinflammation by primarily targeting activated macrophages and partially modulating the functions of age-related interferon-responsive microglia in the brain. Thus, our findings highlight Exo-srIκB as a potential therapeutic agent for treating age-related neuroinflammation.

## Exosome therapy targets neuroinflammation in aging brains

As we age, our bodies undergo changes that can lead to diseases like Alzheimer’s and Parkinson’s. A key factor in this process is inflammation in the brain, driven by a protein called NF-κB. Researcher explored a new way to reduce this inflammation using tiny particles called exosomes. These exosomes were engineered to carry a special form of a protein that blocks NF-κB, called srIκB. In their study, the team injected these exosomes into mice and observed their effects on neuroinflammation. They used advanced techniques to analyze changes in cells of the brain and found that the exosomes reduced inflammation-related genes and altered immune cell behavior. This suggests that the exosomes can help control inflammation in the aging brain. The results indicate that these engineered exosomes could be a promising treatment for age-related brain diseases.

This summary was initially drafted using artificial intelligence, then revised and fact-checked by the author.

## Introduction

Aging is characterized by a gradual decrease in function across multiple organ systems, causing progressive deterioration that eventually results in tissue dysfunction^1^. Aging causes changes in the brain size, vasculature, and cognition. It is considered a major risk factor for most common neurodegenerative diseases, including mild cognitive impairment, Alzheimer’s disease, cerebrovascular disease, and Parkinson’s disease. Many studies have shown that most of the phenotypic characteristics observed in the aging process are the result of a low-grade chronic proinflammatory status called “inflammaging”, which is characterized by increased production of proinflammatory cytokines, acute-phase proteins, reactive oxygen species, and autoantibodies^2^. With aging, the numbers of senescent microglia and infiltrating immune cells in the brain parenchyma increase, exacerbating brain tissue and neuronal cell damage and leading to neurodegeneration. Age-related neuroinflammation is largely induced by increased production of proinflammatory cytokines from senescent and activated microglia, and nuclear factor kappa B (NF-κB) is a critical regulator of microglial proinflammatory cytokine secretion^3^. Many studies have analyzed changes in the microglial phenotype and function with aging and in individuals with neurodegenerative disorders through single-cell RNA sequencing (scRNA-seq)^4–6^; however, currently, therapeutic strategies for modulating microglial activation are unavailable.

The transcription factor NF-κB has received considerable scientific attention over the past few decades as a key factor in immune modulation, inflammation, apoptosis, and neuronal–glial interactions^7^. Excessive activation of the NF-κB signaling pathway is associated with the pathogenesis of several chronic inflammatory disorders and acute inflammation^8^. Owing to the importance of NF-κB, more than 700 NF-κB inhibitors have been developed; however, thus far, no drugs have been approved for human use^9,10^. For the nuclear translocation and activation of NF-κB, the inhibitor of NF-κB (IκB) is phosphorylated by IκB kinases at two conserved serine residues in the N-terminus (Ser32 and Ser36), ubiquitinated, and degraded by the 26S proteasome^11^. Since IκB degradation is the key step in NF-κB activation, genetic constructs expressing an engineered IκB protein, super-repressor IκB (srIκB), which is mutated at specific phosphorylation sites (Ser32 and Ser36 replaced with Ala), result in the formation of a nondegradable, dominant active form of IκB with a prolonged half-life, providing a stable cytoplasmic pool of IκB and thereby preventing NF-κB activation.

Recently, exosomes have been recognized as potent therapeutics or next-generation drug delivery systems for delivering proteins and nucleic acids to target cells^12^. The nonimmunogenic nature of these nanovesicles enables them to protect their cargo from serum proteases and immune responses^13^. Previously, we developed engineered exosomes loaded with srIκB (Exo-srIκB) based on the optogenetically engineered exosome technology “exosomes for protein loading via optically reversible protein‒protein interactions” (EXPLOR) and tested its effects on models of several inflammatory diseases, such as sepsis, acute kidney injury, and preterm birth^10,14–17^. In the present study, we investigated whether Exo-srIκB is effective at alleviating low-grade, chronic neuroinflammation associated with aging, the target cells of these exosomes, and their potential utility for treating age-related neurodegenerative diseases.

## Materials and methods

### Animal experiments

All animals were housed and maintained in a pathogen-free environment/barrier facility; the experiments were conducted according to the international guidelines for the ethical use of experimental animals and were approved by the Institutional Animal Care and Use Committee of the Ewha University College of Medicine (IACUC No. 17-0378). For bulk RNA sequencing, C57BL/6 mice aged 3 months (young) and 15 months (old) were used. Exosomes were administered via intravenous (IV) injections into the lateral tail veins of young female mice (2–3 months old) and old female mice (18–22 months old) daily for 3 days at a dose of 1 × 10^10^ particles per 0.1 mL of vehicle (Exo-Naïve) or Exo-srIκB. Brain tissues were collected from the four groups 20 h after the last injection of exosomes and then analyzed by western blotting, assessments of cytokine and chemokine levels, immunohistochemistry, and scRNA-seq analyses. The Exo-srIκB dose was chosen based on another study in which the same substance was injected intraperitoneally to produce anti-inflammatory effects^17^.

### Tissue preparation and immunohistochemistry

The mice were anesthetized via the intraperitoneal injection of 12.5 mg/kg zolazepam^18^. The tissues were first perfused through the heart with 0.01 M phosphate-buffered saline (PBS, pH 7.4) to remove the blood. The brain tissues were then collected and immediately stored at –80 °C for molecular analysis. Additionally, the tissues were perfused with 4% paraformaldehyde (PFA) in 0.01 M PBS for immunohistochemical analysis. The brain tissues were removed and immersed in fixative overnight at 4 °C. Following fixation, the brain tissues were dehydrated and embedded in paraffin for further processing. Paraffin-embedded tissue blocks were sectioned into 3-µm-thick slices and stained with DAB and target-specific antibodies, followed by analysis under either an Olympus VS200 slide scanner or a BX-50 light microscope (Olympus, Japan). The detailed procedures are described in the Supplementary Information.

### Proteomic profiling of the mouse XL cytokine array

The cortical tissue lysates from wild-type mice (3 and 15 months old) and Exo-Naïve/Exo-srIκB-treated mice (19 months old) were analyzed using a Mouse XL Cytokine Array Kit (R&D Systems, cat. no. ARY028) according to the manufacturer’s instructions. Briefly, the membranes were blocked with blocking buffer for 1 h, incubated with each sample (200 µg) overnight at 4 °C, and then preincubated for 1 h with a biotinylated detection antibody cocktail. The membranes were subsequently incubated with streptavidin-HRP for 30 min and washed with wash buffer. The levels of cytokines/chemokines were measured by determining the spot intensity using ImageJ software and visualized with Morpheus (https://software.broadinstitute.org/morpheus).

### Bulk RNA sequencing and data analysis

Total RNA was extracted from the cerebral cortex of young (3 month-old, *n* = 6 (4 females, 2 males)) and aged (15 month-old, *n* = 7 (3 females, 4 males)) mice. RNA sequencing was performed by Macrogen (Korea). The detailed procedures are described in the Supplementary Information.

The transcriptomic analysis of the bulk RNA sequencing data was performed using R (version 4.2.1). Differentially expressed genes (DEGs) were identified using the R package DESeq2 (version 1.38.3)^19^. Significant DEGs were defined as those demonstrating absolute log2-fold changes in expression > 2 and adjusted *p*-values (Benjamini‒Hochberg–corrected) < 0.05. Gene Ontology (GO) enrichment analysis and gene set enrichment analysis (GSEA) of significant DEGs were performed using the R package clusterProfiler (version 4.6.2)^20^; GO terms and gene sets with adjusted *p*-values (Benjamini‒Hochberg-corrected) < 0.05 were considered statistically significant. Gene set variation analysis (GSVA) was performed using the R package GSVA (version 1.46.0)^21^; gene sets with adjusted *p*-values (Benjamini‒Hochberg-corrected) < 0.05 were deemed to be significantly enriched.

### Exosome production and characteristics

Exosomes (Exo-Naïve: lot. 200P2203, Exo-srIκB: lot. FFM-21-047) were produced by ILIAS Biologics Inc. (Korea) through upstream and downstream manufacturing processes. Cell cultivation was performed for 4 days (as upstream processes) using the WAVE method with blue light exposure. Next, two ultrafiltration and two column purification steps were performed (as downstream processes)^10,22^. The quality of the purified exosomes was assessed by determining the particle number and size using the nanoparticle tracking analysis (NTA) method, and the presence of major positive and negative markers of the exosomes was confirmed via western blotting, as described previously^14,23,24^. The detailed procedures are described in the Supplementary Information.

### Single-cell RNA-seq data analysis

For the single-cell RNA sequencing (scRNA-seq) study, four young female mice (2–3 months old) and four old female mice (18–22 months old) were included. Each group consisted of two Exo-Naïve-treated mice and two Exo-srIκB-treated mice. scRNA-seq was performed by ROKIT Genomics (Korea). The detailed procedures are described in the Supplementary Information.

All bioinformatic analyses were performed using R (version 4.2.1). Initially, the cells were retained based on the following criteria: a number of detected genes between 250 and 8000, a number of UMI counts between 500 and 100,000, a novelty score (defined as the log10 of the number of detected genes divided by the log10 of the number of UMI counts) above 0.8, and < 20% mitochondrial UMIs. Data from different groups were subsequently independently normalized using SCTransform^25^ while the ‘mitochondrial percentage’ was regressed out, which was implemented in the Seurat pipeline^21^. The normalized data were then integrated using the canonical correlation analysis (CCA) method^26^ following the steps tailored for SCTransform-normalized datasets provided in Seurat. The top 15 principal components (PCs) were selected for the downstream analysis. Clustering analysis was performed using the Louvain algorithm, and the clusters were visualized using the uniform manifold approximation (UMAP) method. Initial cluster annotations were based on their transcriptomic profiles and the expression of known canonical markers for each lineage (*Sox10, Olig1*, and *Olig2* for the oligodendrocyte lineage; *Gja1* and *Cldn10* for the astrocyte lineage; *Snap25* for the neuronal lineage; *Dynlrb2* for ependymal cells; *Esam* and *Myl9* for cells of the vasculature; and *C1qc, Cd57, Cx3cr1*, and *Ccr2* for immune cells)^27^.

A subclustering analysis was performed by extracting each lineage and reclustering the data using the same strategy. However, a few clusters that appeared to be oversegmented based on heuristic criteria (such as the absence of unique DEGs between neighboring clusters, significant overlap in the UMAP plot between neighboring clusters, or the presence of clusters in which a small number of cells were mixed with those from a cluster with larger cell numbers in the UMAP plot) were merged manually. Subcluster annotations were determined by comparing the cluster marker genes identified using the FindMarkers() function in Seurat with well-known cell type-specific marker genes (e.g., *Cspg4* and *Bmp4* for OPCs; *Cldn11, Opalin*, and *Klk6* for mtOLGs; *Npy* for OEGs; *Cd44* for ARPs; *Slc1a3, S100b*, and *Mfge8* for ASCs; *Dynlrb2* and *Ccdc153* for EPCs; *Esam* and *Cd34* for ECs; *Myl9, Kcnj8*, and *Acta2* for PCs; *Ptgds* and *Il33* for VLMCs; *Col1a1* and *Col1a2* for fibroblasts; *Aif1, Itgam, P2ry12, Tyrobp*, and *Cx3cr1* for microglia; *F13a1* and *Mrc1* for infiltrated macrophages; *Cd19* and *Ms4a1* for B cells; *Cd3e, Cd4, Cd8a*, and *Foxp3* for T cells; *Gad1* for InhN; and *Cpne4, Tshz2*, and *Kcnh4* for ExcN)^4,6,27–31^.

### Calculation of scores

Scores were calculated using the AddModuleScore() function in Seurat, which computes the average expression levels of each set of genes at the single-cell level, subtracted from the aggregated expression of background genes. All computed sets of genes were binned based on their average expression; the control background genes were randomly selected from each bin. The detailed procedures are described in the Supplementary Information.

### Differential expression analysis

We performed a differential expression analysis across various conditions using the raw UMI count matrix derived from single-cell data with the R package DESeq2 (version 1.38.3)^19^. We optimized the single-cell data analysis using the likelihood ratio test (test = “LRT”) and set the parameters as described previously^32–34^.

### Trajectory analysis

We used Monocle 3 (version 1.3.1) to examine the transition of macrophage and oligodendrocyte subsets^35^. The learn_graph(), order_cells(), and plot_cells() functions were employed to learn the trajectories and map the differentiation of different macrophage or oligodendrocyte subtypes. Genes whose expression levels changed significantly along a trajectory within macrophages were identified using the graph_test() function with default parameters, with the exception of neighbor_graph, which was set as “principal_graph”; genes with FDR-corrected *q*-values < 0.001 were considered significant. Modules of genes showing significant changes in expression along a trajectory were identified using the find_gene_modules() function (resolution = 0.01).

### SCENIC analysis

We conducted single-cell regulatory network inference and clustering (SCENIC) analysis of microglia and macrophages in the brain using the pySCENIC package (a Python implementation of the SCENIC pipeline, version 0.12.1)^36^ to assess alterations in transcriptional regulatory networks induced by aging and Exo-srIκB administration. First, the normalized expression count matrix was filtered to maintain genes expressed in at least 1% of the cells. Next, transcription factor–target gene coexpression modules were identified using the GRNbosst2 algorithm. Within these identified modules, regulons were determined using the motif annotation and the cisTarget database for mice provided by SCENIC, with the default parameters (motif annotation: motifs-v9-nr.mgi-m0.001-o0.0.tbl; cisTarget database: mm10_10kbp_up_10kbp_down_full_tx_v10_clust.genes_vs_motifs.rankings.feather). Finally, regulon activities were calculated in each single cell using the AUCell method, and the resulting AUCell value matrix was integrated into the Seurat object.

### Cell–cell communication analysis

We used CellChat (version 1.6.1) to analyze cell–cell communication among immune cells^37^. First, we identified the interactions between overexpressed ligands and receptors in each group using the secreted signaling database from CellChatDB.mouse. We subsequently examined the signaling networks with larger differences based on their Euclidean distances in the shared two-dimensional space according to the functional similarity. Finally, we employed the netVisual_bubble() function to identify significant changes in ligand–receptor interactions from microglia or macrophages (sources.use) to T cells or B cells (targets.use) by comparing the communication probabilities between the specific two groups.

### Cell culture and treatment

The RAW264.7 cell line was purchased from the American Type Culture Collection (ATCC; Manassas, VA, USA). EL4 and FB2 cell lines were obtained from the Korean Cell Line Bank (KCLB; Korea). RAW264.7 macrophages and FB2 cells were maintained in RPMI 1640 medium, and EL4 cells were cultured with Dulbecco’s modified Eagle’s medium (DMEM; WelGENE Inc.) supplemented with 10% FBS and 1% penicillin/streptomycin (Gibco). RAW264.7 macrophages were seeded in 60 mm culture dishes at a density of 1 × 10^6^ cells and allowed to attach overnight. Next, the cells were stimulated with 1 µg/mL lipopolysaccharide (LPS; Sigma‒Aldrich, cat. no. L2880) (or left unstimulated) and treated with 1 × 10^7^ Exo-srIκB particles in RPMI 1640 medium containing 1% FBS for 24 h. The conditioned media were collected and then used to treat EL4 or FB2 cells seeded in 6-well plates at a density of 5 × 10^5^ cells/well for 24 h. Relative mRNA levels were determined using quantitative real-time PCR (qRT‒PCR), and the detailed procedures are described in the Supplementary Information.

### Statistical analyses

Statistical analyses were performed using Student’s *t-*test to compare the differences between samples from two groups. For comparisons of multiple groups, one-way ANOVA was used with Bonferroni’s or Tukey’s correction as a post hoc test. A Pearson chi-square test was performed to determine the statistical significance of the differences in cell type composition across the four conditions. All analyses were conducted using Prism 5.0 software (GraphPad Software, USA) or R (version 4.2.1), with statistical significance set at *p* < 0.05.

## Results

### Increased inflammatory responses in aged mouse brains

**Fig. 1:**
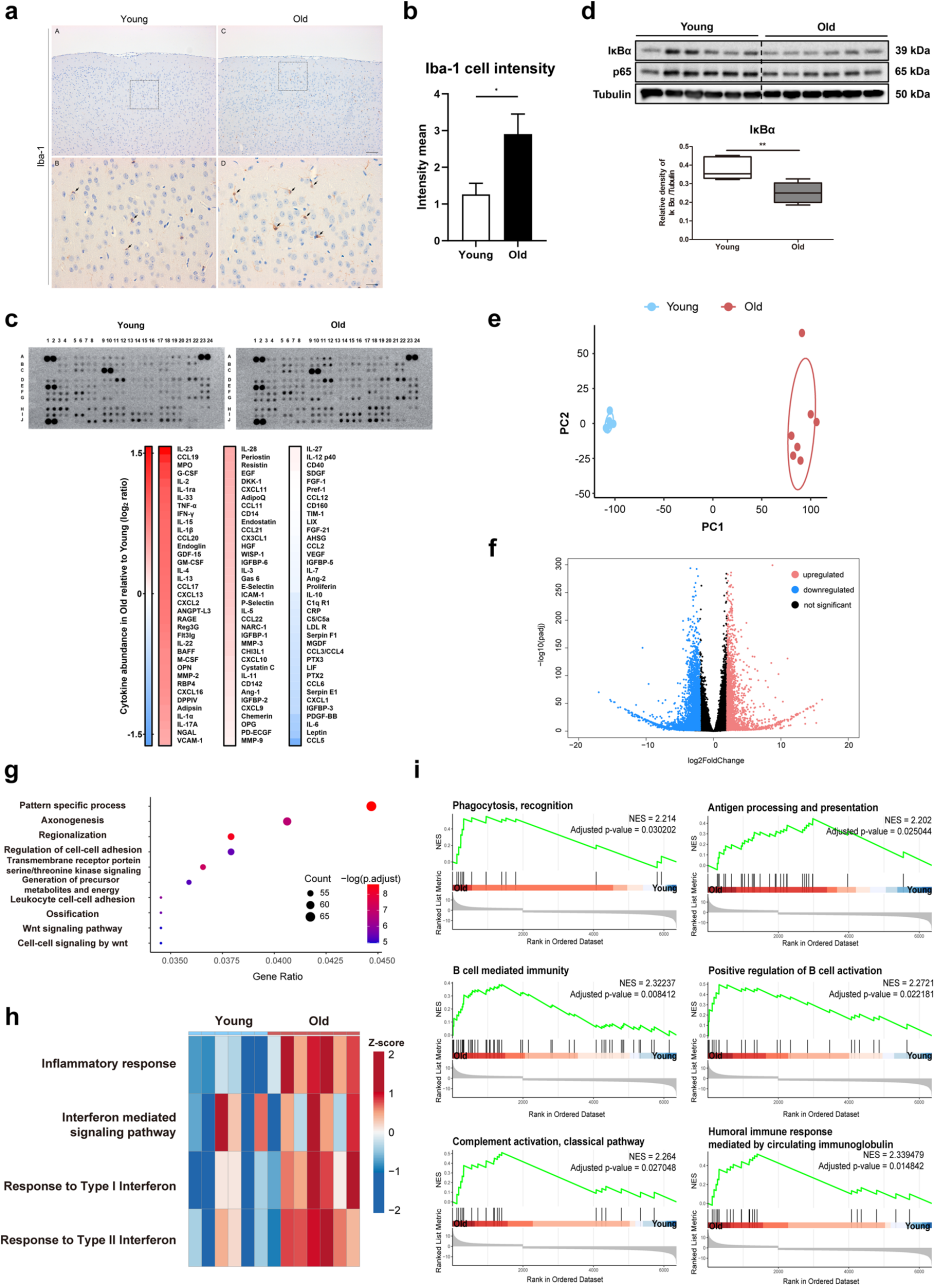
Increased inflammatory responses in aged mouse brains. **a** Immunohistochemical staining for Iba-1 in the cerebral cortex of young (2–3 months old) and old (21–22 months old) mice (arrow). Scale bars = 200 µm (A, C) and 50 µm (B, D). **b** The relative intensity of Iba-1-immunopositive cells in the brains of young and old mice was quantified (*n* = 6, means ± SDs, *p-value < 0.05). **c** Cytokine array results for the cerebral cortex of young (3 months old) and old (15 months old) mice. The relative expression of the protein spots detected by the array is presented in a graph based on a comparison of the signal intensities between young and old mice. **d** Immunoblots showing the levels of IκBα and p65 in young and old mice (*n* = 6). Tubulin was used as the loading control. The graph presents the intensities of IκBα normalized to those of tubulin (means ± SDs, **p-value < 0.01). **e**–**i** Bulk RNA sequencing of the cerebral cortex of young (3 month-old, *n* = 6 (4 females, 2 males)) and old (15 month-old, *n* = 7 (3 females, 4 males)) mice. **e** Principal component analysis (PCA) plot of the RNA-seq data obtained from the brains of young and old mice. **f** Volcano plot showing −log10 (adjusted *p*-value) and log2 (fold change) values for all genes, with those that were significantly upregulated in old mice (Benjamini‒Hochberg (BH)-adjusted *p*-value < 0.05 and log2 fold change > 2) indicated by red highlighting, and those that are significantly downregulated in old mice (BH-adjusted *p*-value < 0.05 and log2 fold change < −2) indicated by blue highlighting. **g** Gene Ontology (GO) terms enriched among significantly upregulated differentially expressed genes (DEGs) in old mice. The dot color and size represent the *p*-value and gene ratio (gene count in a specific term divided by the total number of genes), respectively. The top 10 GO terms, according to the gene ratio with BH-adjusted *p*-values < 0.05, are listed. **h** Heatmap showing the results of gene set variation analysis (GSVA). The color represents the Z scored and normalized enrichment score. **i** Gene set enrichment analysis (GSEA) of GO biological processes for significant DEGs. Significant GO terms (BH-adjusted *p-*value < 0.05) involved in the immune response are presented.

Previously, we reported that aging induces changes in brain endothelial cells, leading to leukocyte infiltration into the brain and age-associated neuroinflammation^18^. To examine the changes in protein levels or cell numbers, a histological analysis of the brain tissues was performed to detect ionized calcium-binding adapter molecule 1 (Iba-1)-positive cells in young (2–3 month-old) and aged (21–22 month-old) mice. The number of Iba-1-positive cells was greater in the aged mouse brain than in the young mouse brain (Fig. 1a, b). Furthermore, the expression of 79 chemokines, including interleukin (IL)-23, C-C motif ligand (CCL) 19, IL-2, and IL-15 (out of 111 chemokines tested), was upregulated in the brains of old mice compared with those of young mice (Fig. 1c). By analyzing the transcriptional regulation of these chemokines using the TFLink gateway, we found that one of the key transcription factors responsible for the increased chemokine levels was NF-κB; therefore, we further analyzed the total IκBα and p65 expression levels using an immunoblot analysis. The total IκBα protein level was significantly lower in the old mouse brain than in the young mouse brain (Fig. 1d), indicating that the NF-κB pathway is activated in the aged brain. We performed bulk RNA sequencing of the cerebral cortex from young and aged mice to understand age-related changes at the transcriptome level. The quality assessment of the bulk RNA sequencing data was satisfactory, with no clear outliers after normalization; the correlation analysis demonstrated a high degree of correlation among the biological replicates (Supplementary Fig. 1a, b). Principal component analysis (PCA) revealed that age significantly influenced the transcriptome pattern of the brain, but sex did not (Fig. 1e, Supplementary Fig. 2). Through a differential expression analysis of the brains of old and young mice, we identified 6354 significant DEGs (1992 upregulated and 4362 downregulated), indicating distinct gene expression patterns between the two groups (Fig. 1f). The GO enrichment analysis of the significant DEGs revealed that the GO terms enriched with the upregulated DEGs were related to the regulation of cell–cell adhesion and leukocyte cell–cell adhesion, which may contribute to age-related inflammation (Fig. 1g). Furthermore, GSVA revealed that the inflammatory response and interferon-related signaling pathways were coordinately upregulated in the brains of old mice (Fig. 1h). Additionally, GSEA confirmed that the expression levels of significant DEGs in the brains of old mice were positively correlated with numerous inflammation-related GO terms, such as phagocytosis, antigen processing and presentation, B-cell-mediated immunity, positive regulation of B-cell activation, the classical complement activation pathway, and the humoral immune response mediated by circulating immunoglobulins (Fig. 1i).

### Single-cell transcriptomic profiling of brains from mice treated with Exo-srIκB or Exo-Naïve

**Fig. 2:**
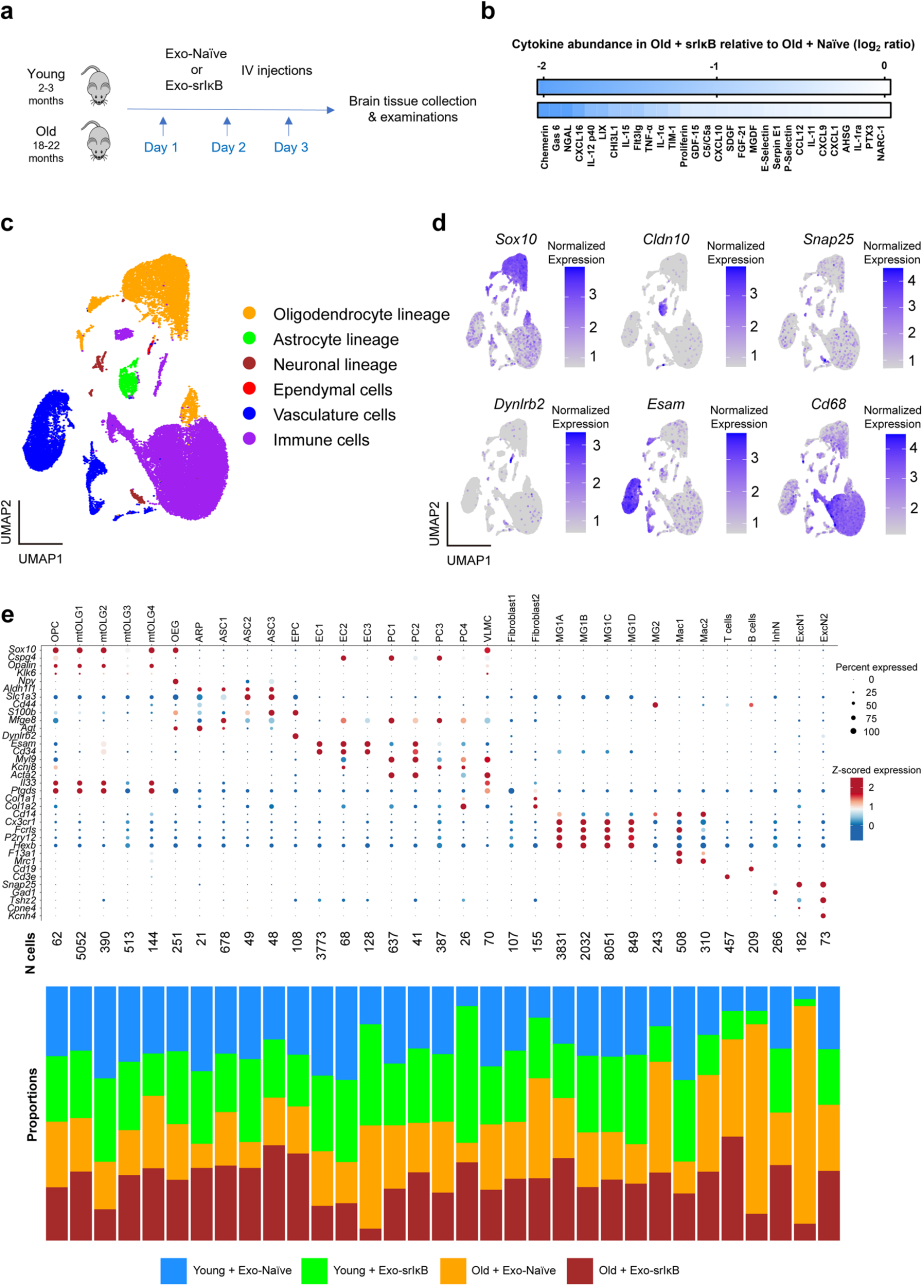
Single-cell transcriptomic analysis of brains from mice in the four different groups. **a** Experimental protocol. **b** Cytokine array results for the cerebral cortex of old Exo-Naïve-treated and Exo-srIκB-treated mice. Heatmap of the relative expression of the protein spots detected by the array based on a comparison of the signal intensities between old Exo-Naïve-treated and Exo-srIκB-treated mice. **c** Single-cell RNA sequencing (scRNA-seq) of whole brains obtained from Exo-Naïve/Exo-srIκB-treated young (2–3 months old, *n* = 2 per group) and old (18–22 months old, *n* = 2 per group) female mice. Uniform manifold approximation (UMAP) plots of cells (*n* = 29,719) from all groups. The cells are colored based on their respective lineages. **d** UMAP visualization of each lineage showing the expression of well-known representative marker genes. **e** (Top panel) Dot plot of representative marker genes for molecularly identified cell types. The dot size and color represent the percentage of cells expressing specific marker genes within each cell type and Z scores for normalized expression values, respectively. (Bottom panel) Bar plots showing the proportion of cells per cluster across the different groups.

Young (2–3 months old) or old (18–22 months old) mice were administered exosomes containing super-repressor IκB (Exo-srIκB) or control exosomes (Exo-Naïve) to investigate the effect of NF-κB inhibition on aging-related neuroinflammation. The production of Exo-Naïve (nonengineered exosomes derived from HEK293F cells, vehicle) and Exo-srIκB (engineered exosomes) was conducted as previously described^22^, and the mice were intravenously administered 1 × 10^10^ particles per 0.1 mL of Exo-Naïve or Exo-srIκB for three consecutive days (Fig. 2a). We used NTA to measure exosome size and concentration (Supplementary Fig. 3a). Exo-Naïve did not contain payload proteins, such as srIκB, CRY2, and CD9 (CD9-CIBN), but expressed positive markers for exosomes. The exosomes did not express the cell organelle marker GM130 (Supplementary Fig. 3b). Twenty hours after the third exosome injection, brain tissues were collected and analyzed.

First, the cerebral cortex of the Exo-srIκB-treated and Exo-Naïve-treated old mice was analyzed using a cytokine/chemokine array (Fig. 2b). In old mice treated with Exo-srlκB, we noted reduced levels of 35 proteins (among the 79 proteins whose levels increased), including IL-1α, IL-11, and IL-15 (Fig. 1c). Additionally, histological measurements of Iba-1-positive cells from the four groups were performed (Supplementary Fig. 4). The number of Iba-1-positive cells in the cortex was greater in the old mice than in the young mice (Fig. 1a). The changes in the numbers of Iba-1-positive cells in the cerebral cortex and hippocampus of the Exo-Naïve- and Exo-srIκB-treated old mice were not statistically significant.

Next, we conducted scRNA-seq of whole mouse brains from two young Exo-Naïve-treated mice, two young Exo-srIκB-treated mice, two old Exo-Naïve-treated mice, and two old Exo-srIκB-treated mice to investigate the effects of Exo-srIκB on various cell types in the mouse brain. We assessed the quality control metrics of the data (Supplementary Fig. 5) and filtered out low-quality cells; 29,719 cells were available for the downstream analysis. Among these cells, 7977 were obtained from Exo-Naïve-treated young mice, 8008 from Exo-srIκB-treated young mice, 6610 from Exo-Naïve-treated old mice, and 7124 from Exo-srIκB-treated old mice. Following normalization, integration, and clustering of the gene expression matrix with Seurat^21^, we initially annotated the cell types into six lineages based on their transcriptomic profiles and the expression of known canonical marker genes for each lineage (Fig. 2c, d). After the initial annotation, we conducted subclustering within each lineage to achieve a high-resolution overview of the data; we obtained 33 distinct cell types (Fig. 2e): oligodendrocyte precursor cells (OPCs), mature oligodendrocytes (mtOLG1–mtOLG4), olfactory ensheathing glia (OEGs), astrocyte restricted precursor (ARP) cells, mature astrocytes (ASC1–ASC3), ependymal cells (EPCs), endothelial cells (EC1–EC3), pericytes (PC1–PC4), vascular leptomeningeal cells (VLMCs), fibroblasts (Fibroblast1–Fibroblast2), microglia (MG1A–MG2), infiltrated macrophages (Mac1–Mac2), T cells, B cells, inhibitory neurons (InhN), and excitatory neurons (ExcN1–ExcN2). The neuronal lineage was poorly captured with low diversity, probably due to the single-cell dissociation procedures for mammalian adult brain tissues^38^. However, since Exo-srIκB is expected to target primarily immune cells and immune cells were captured with high resolution in our data (16,490 cells), we conducted a downstream analysis that focused mainly on immune cell populations.

### Changes in the cell type and cell state composition after Exo-srIκB treatment

**Fig. 3:**
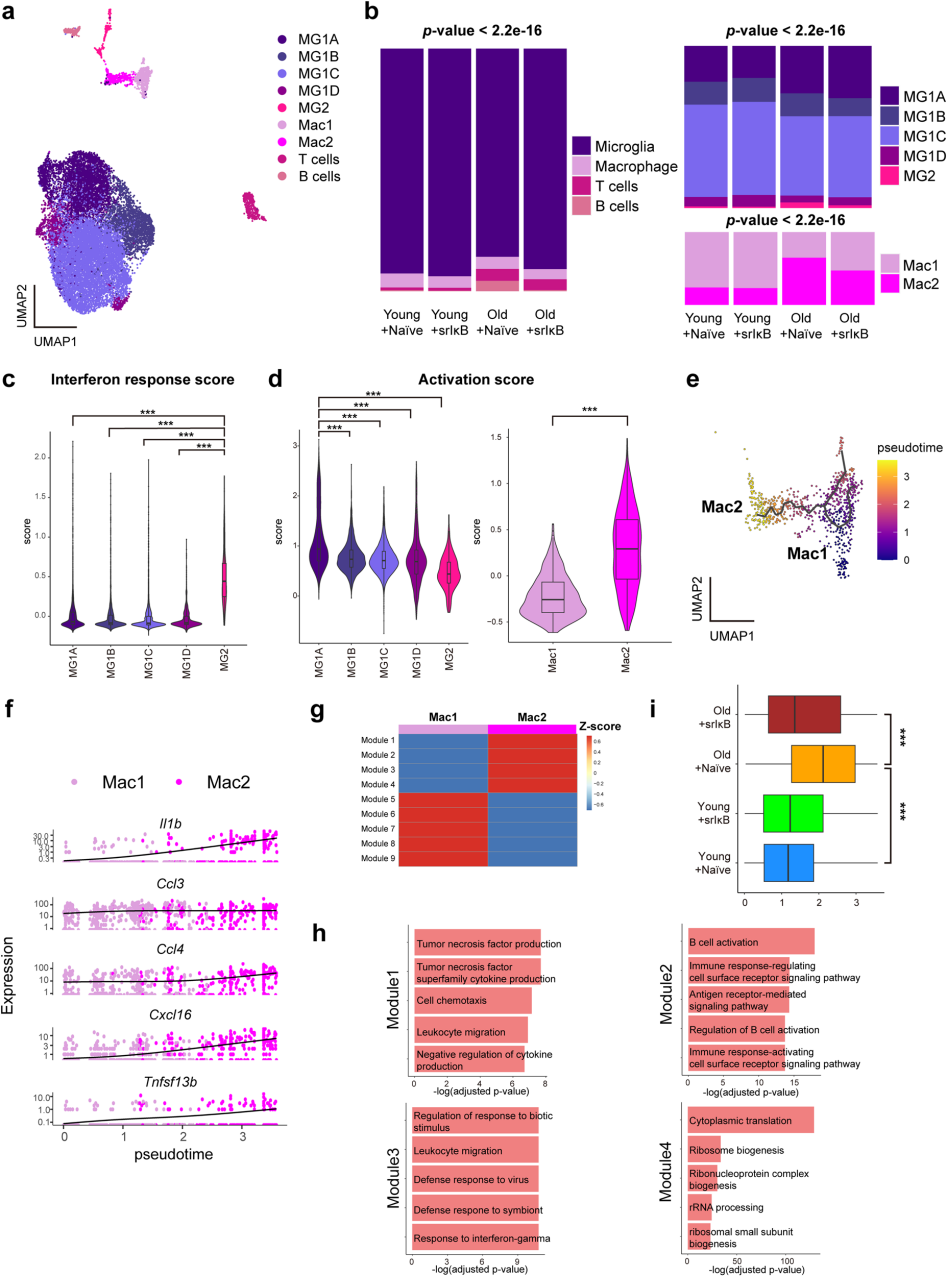
Changes in the cell type and cell state composition of immune cells were observed across the four different groups. **a** Uniform manifold approximation (UMAP) plot of immune cells (*n* = 16,490). **b** Proportions of cell types (left panel) among immune cells and cell states (right panel) among microglia or macrophages across the four groups. A chi-square test was conducted to determine *p*-values. **c**, **d** Violin plots showing the interferon response scores (**c**) and activation scores (**d**) among the different states of microglia or macrophages. *** indicates an adjusted *p*-value < 0.001 (one-way ANOVA with Bonferroni’s multiple comparison correction) between two compared groups. **e** UMAP plot with the trajectories of macrophages, colored based on the pseudotime. **f** Changes in the expression levels of selected genes within macrophages across the pseudotime axis. Individual dots represent individual cells, which are colored based on the corresponding cell state, and black lines represent trends in the expression levels of selected genes. **g** Heatmap presenting the identified modules of genes whose expression changed significantly along the trajectory of macrophages. The indicated coloration represents normalized, aggregated, and Z scores for the expression levels of specific modules. **h** Bar plots present the top 5 significantly enriched Gene Ontology (GO) terms within each module. GO terms with Benjamini‒Hochberg adjusted *p*-values < 0.05 were considered significant, and the terms were ordered by −log10 (adjusted *p*-value). **i** Box plots represent the distribution of the pseudotime of macrophages across four different groups. *** indicates an adjusted *p*-value < 0.001 (one-way ANOVA with Bonferroni’s multiple comparison correction) between two compared groups.

Subclustering of immune cells revealed four distinct cell types (microglia, macrophages, T cells, and B cells) with diverse cell states (e.g., microglia: MG1A, MG1B, MG1C, MG1D, and MG2; macrophages: Mac1 and Mac2) (Fig. 3a). The composition of immune cell types significantly changed across the four groups, with increases in T and B-cell numbers in old Exo-Naïve-treated mice and a decrease in B-cell numbers in old Exo-srIκB-treated mice (Fig. 3b, left panel). Furthermore, when we compared the composition of different cell states within the microglia, we observed increases in the MG1A and MG2 proportions in the old Exo-Naïve-treated mice compared with those in the young Exo-Naïve-treated mice. The composition of cell states within macrophages also changed, with an increase in Mac2 numbers in old Exo-Naïve-treated mice compared with young Exo-Naïve-treated mice (Fig. 3b, right).

Several studies have reported that interferon-responsive microglia and activated macrophages play prominent roles in the aged brain^6,39,40^. Therefore, we calculated the interferon response and activation scores of microglia and macrophages. The interferon response score was significantly higher in the age-related state MG2, a cell state whose proportion decreased following Exo-srIκB treatment in old mice, than in the old Exo-Naïve-treated mice (Fig. 3b, c). Additionally, the age-related clusters MG1A and Mac2 presented significantly higher activation scores than the other states did (Fig. 3d); Exo-srIκB decreased the proportion of Mac2 macrophages in old mice (Fig. 3b, right panel).

Next, we examined the trajectories of the two macrophage states (Mac1 and Mac2) to investigate their transdifferentiation. We observed that Mac1 gradually transitioned into age-related Mac2 with pseudotime (Fig. 3e). Moreover, some proinflammatory cytokines and chemokines, such as *Il1b, Ccl3, Ccl4, Cxcl16*, and *Tnfsf13b*, were gradually upregulated along the trajectory, which was consistent with the enrichment of activation-related genes in the Mac2 state (Fig. 3f). Additionally, we identified four gene modules whose expression increased significantly with pseudotime (Fig. 3g). The genes belonging to these four modules were associated with biological processes such as tumor necrosis factor production, leukocyte migration, cell chemotaxis, and the antigen receptor-mediated signaling pathway, indicating that macrophage activation-related cellular processes were gradually activated along pseudotime (Fig. 3h). The distribution of pseudotime trajectories within macrophages across the four groups revealed that macrophages from old Exo-Naïve-treated mice were ordered toward the end of the trajectory; however, they exhibited a significant shift toward the beginning of the trajectory in old Exo-srIκB-treated mice (Fig. 3i).

Additionally, we investigated the transcriptomic differences among oligodendrocytes, astrocytes, and endothelial cells, which are aging-sensitive cell types. Subclustering of the oligodendrocyte lineage yielded six cell types (Supplementary Fig. 6a); a comparison of the composition of these cell types indicated that the mtOLG4 proportions were significantly higher in old Exo-Naïve-treated mice than in young Exo-Naïve-treated mice and subsequently decreased following Exo-srIκB treatment in old mice. Furthermore, the mtOLG2 proportions were significantly lower in old Exo-Naïve-treated mice than in young mice and further decreased in old mouse brains following Exo-srIκB treatment (Supplementary Fig. 6b). The GO enrichment analysis of the marker genes identified in each mature oligodendrocyte cluster revealed that myelination- and axon ensheathment-related processes were active in mtOLG1, whereas migration-related pathways were active in mtOLG2. In contrast, mtOLG3 and mtOLG4 exhibited proinflammatory features (Supplementary Fig. 6c). The trajectory analysis of mature oligodendrocytes revealed that these oligodendrocytes followed linear transitions, starting from mtOLG2 to proinflammatory mtOLG3 (Supplementary Fig. 6d). We calculated the interferon response scores among the four mature oligodendrocyte subtypes to distinguish between mtOLG3 and mtOLG4, which exhibit proinflammatory features, since interferon-responsive oligodendrocytes are known to play a role in brain white matter aging^39^; the age-related cluster mtOLG4 presented the highest score (Supplementary Fig. 6e). Moreover, defects in myelinating oligodendrocytes result in demyelination injury and, eventually, permanent neuronal loss in conditions such as multiple sclerosis, aging, or Alzheimer’s disease^41–44^. We further assessed the myelination-related and demyelination-related signatures in these oligodendrocyte clusters (Supplementary Table 1)^45^. The demyelination-associated signature was significantly prominent in proinflammatory oligodendrocytes (mtOLG3 and mtOLG4) (Supplementary Fig. 6f), and myelination was the most active signature in mtOLG1 (Supplementary Fig. 6g). As biological processes involved in migration and actin filament organization are activated in the initial stages of the response to myelin injury, during which OPCs gradually migrate and later differentiate into myelinating oligodendrocytes, we determined that mtOLG2 represented the initial state of myelinating oligodendrocytes, which later transitioned into a more mature state (mtOLG1). This conclusion was also supported by the trajectory analysis (Supplementary Fig. 6d). In old mice, the proportions of interferon-responsive oligodendrocytes (mtOLG4) increased significantly, and those of initial myelinating oligodendrocytes (mtOLG2) decreased, suggesting prolonged chronic inflammation in the aged brain and resulting defects in the remyelination process in response to the accumulation of myelin injury with aging. After Exo-srIκB treatment, the proportion of mtOLG4 decreased significantly. However, unexpectedly, the mtOLG2 proportion also decreased further. Although the exact mechanisms underlying these processes remain unclear, the decreased need for remyelination due to mitigated neuroinflammation in old Exo-srIκB-treated mice may be one hypothesis.

Subclustering of the astrocyte lineage revealed four cell types (Supplementary Fig. 7a). We identified the ASC3 cluster as activated astrocytes. Although reactive and activated astrocytes are known to emerge and increase in number in the aged brain^6,46^, the proportion of this subtype did not change significantly across the four groups (Supplementary Fig. 7b, c).

Subclustering of vasculature cells revealed ten cell types (Supplementary Fig. 7d). The composition of vasculature cells was highly variable across the four groups. The proportion of EC3 in old Exo-Naïve-treated mice was significantly increased compared with that in young Exo-Naïve-treated mice and decreased in old Exo-srIκB-treated mice (Supplementary Fig. 7e). Interestingly, in EC3, propermeability (*Plvap* and *Fut4*)^47–49^ and immune cell recruitment-related (*Sele* and *Selp*)^50–52^ genes were robustly expressed, whereas blood‒brain barrier transport-related genes (*Mfsd2a*, *Slc16a1*, and *Slc38a5*)^47^ were downregulated (Supplementary Fig. 7f). The GO enrichment analysis further confirmed that in EC3, endothelial and leukocyte migration-related pathways were enriched in the biological processes, whereas transport across the membrane was diminished (Supplementary Fig. 7g, h). Collectively, these results present dynamic changes in the composition of brain endothelial cells in response to aging and Exo-srIκB treatment, characterized by the enrichment of more permeable endothelial cells with aging and the subsequent decrease in their proportions following Exo-srIκB administration.

### Changes in the molecular signatures and cellular processes of individual cell types across the four different groups

**Fig. 4:**
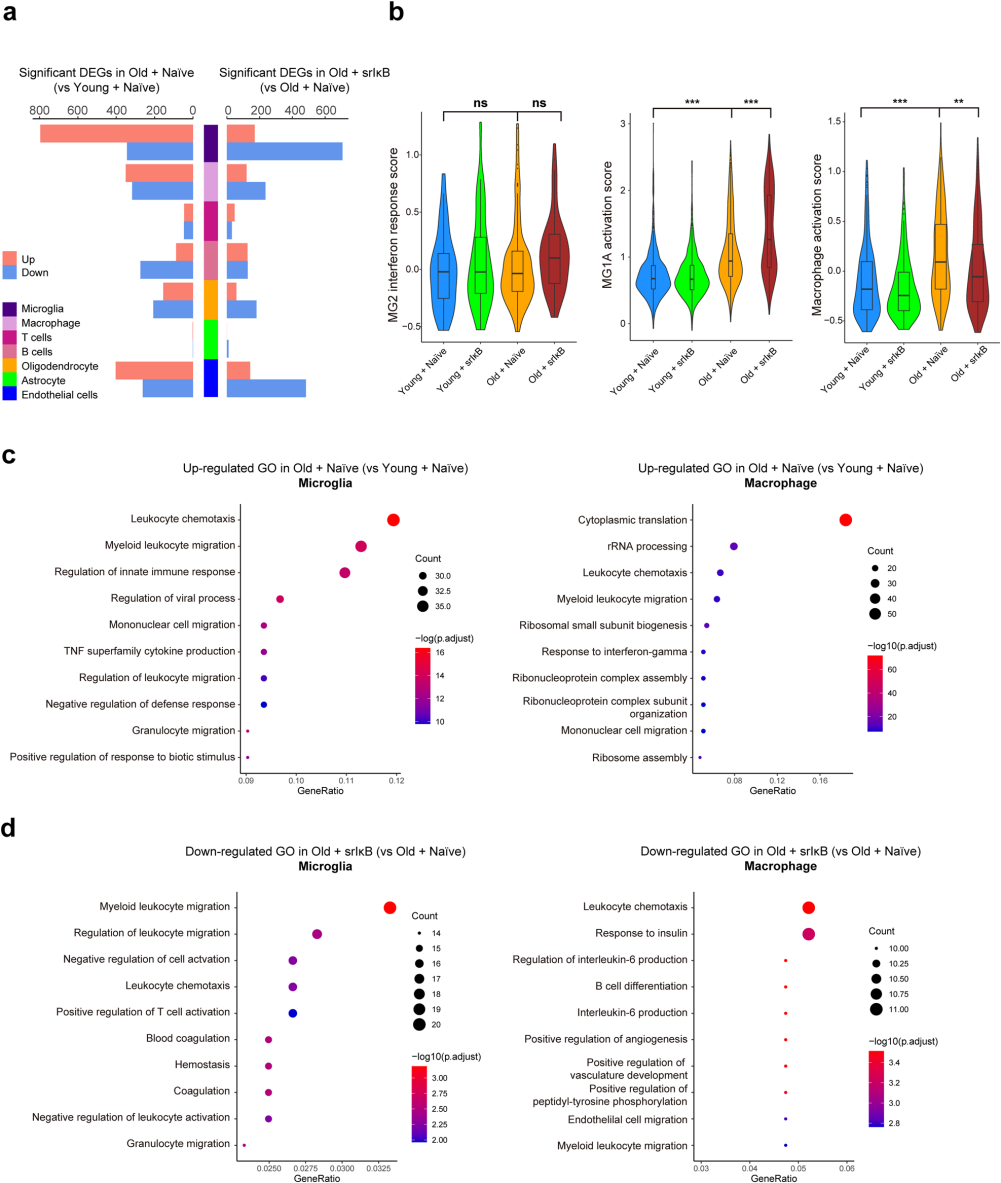
Changes in the molecular signatures and cellular processes of microglia and macrophages across the four different groups. **a** Number of significant DEGs between Exo-Naïve-treated old vs. young mice and Exo-srIκB-treated vs. Exo-Naïve-treated old mice in each immune cell type. Significant DEGs were defined as genes with a Benjamini‒Hochberg (BH)-adjusted *p*-value < 0.05 and an absolute value of log2-fold change  > 0.5 between the two compared groups. **b** Violin plots display the scores of selected molecular signatures across the four groups. The interferon response score was calculated within MG2. The microglial activation score was calculated for MG1A. The macrophage activation score was calculated for all states of macrophages. *** indicates an adjusted *p*-value < 0.001, and ns indicates no statistical significance (one-way ANOVA with Bonferroni’s multiple comparison correction) between two compared groups. **c** Gene Ontology (GO) terms that were enriched among the significantly upregulated DEGs in the microglia and macrophages from the old Exo-Naïve-treated mice compared with those in the microglia and macrophages from the young Exo-Naïve-treated mice. The dot color and size represent the *p*-value and gene ratio, respectively. The top 10 GO terms, according to the gene ratio with BH-adjusted *p*-values < 0.05, are listed. **d** GO terms that were enriched among significantly downregulated DEGs in microglia and macrophages from old Exo-srIκB-treated mice compared with those from old Exo-Naïve-treated mice. The top 10 GO terms, according to the gene ratio with BH-adjusted *p*-values < 0.05, are listed.

With aging, more DEGs were upregulated in microglia, brain-infiltrating macrophages, and endothelial cells than in other cell types. Following Exo-srIκB treatment in old mice, these cell types presented more downregulated DEGs than other cell types did (Fig. 4a).

We further examined the changes in the interferon response scores and activation scores of microglia and macrophages (Fig. 4b). Although the proportion of interferon-responsive MG2 increased in the brains of old mice but decreased after Exo-srIκB treatment, the interferon response scores of MG2 under the four conditions did not differ significantly. The microglial activation score of MG1A increased in old Exo-Naïve-treated mice compared with that in young Exo-Naïve-treated mice. However, unexpectedly, this score increased further in old mice after Exo-srIκB treatment. Given that the proportion of MG1A did not decrease after Exo-srIκB administration in old mice (Fig. 3b), these results suggest that MG1A did not effectively respond to Exo-srIκB both quantitatively and qualitatively. In contrast, the macrophage activation score increased in the old Exo-Naïve-treated mice compared with that in the young Exo-Naïve-treated mice and subsequently decreased in the old Exo-srIκB-treated mice compared with that in the old Exo-Naïve-treated mice. We compared the T and B-cell activation scores to investigate whether the activities of T and B cells also changed significantly across the four conditions, but they were not found to vary significantly among the four conditions (Supplementary Fig. 8a, b). Moreover, the interferon response scores for oligodendrocytes and the activation scores for astrocytes did not differ significantly among the four groups (Supplementary Fig. 8c, d). We performed a GO enrichment analysis using significant DEGs among the four conditions within immune cells to more broadly evaluate the cellular processes that changed with aging and Exo-srIκB treatment; the findings revealed that in Exo-Naïve-treated mice, inflammatory and leukocyte activation-related processes were enriched, especially in microglia and macrophages (Fig. 4c, Supplementary Fig. 9). Moreover, compared with those in old Exo-Naïve-treated mice, the downregulated DEGs in old Exo-srIκB-treated mice were enriched in leukocyte migration, chemotaxis, and activation, especially in microglia and macrophages (Fig. 4d, Supplementary Fig. 10).

Next, we investigated the transcriptional networks of immune cells in the brain in response to systemic Exo-srIκB treatment using SCENIC analysis. Our analysis of significantly enriched regulons in each microglial and macrophage subtype revealed that transcription factors (TFs) associated with proinflammatory responses and classical macrophage activation (*Stat4*, *Stat5a*, and *Irf1*)^53–55^ were activated in MG1A, MG2, or Mac2, whereas type I interferon-related TFs (*Stat1*, *Stat2*, *Irf7*, and *Irf9*)^39,56^ exhibited increased activity in MG2. In contrast, NF-κB family TF activity did not differ significantly across the four conditions (Supplementary Fig. 11a, b). Furthermore, the expression levels of *Nfkb1* and *Nfkb2* target genes varied heterogeneously across conditions and cell types (Supplementary Fig. 11c, d, e), suggesting that the overall NF-κB-mediated transcriptional networks did not change significantly under the four conditions.

### Alterations in cell–cell communication among immune cells during aging and Exo-srIκB treatment

**Fig. 5:**
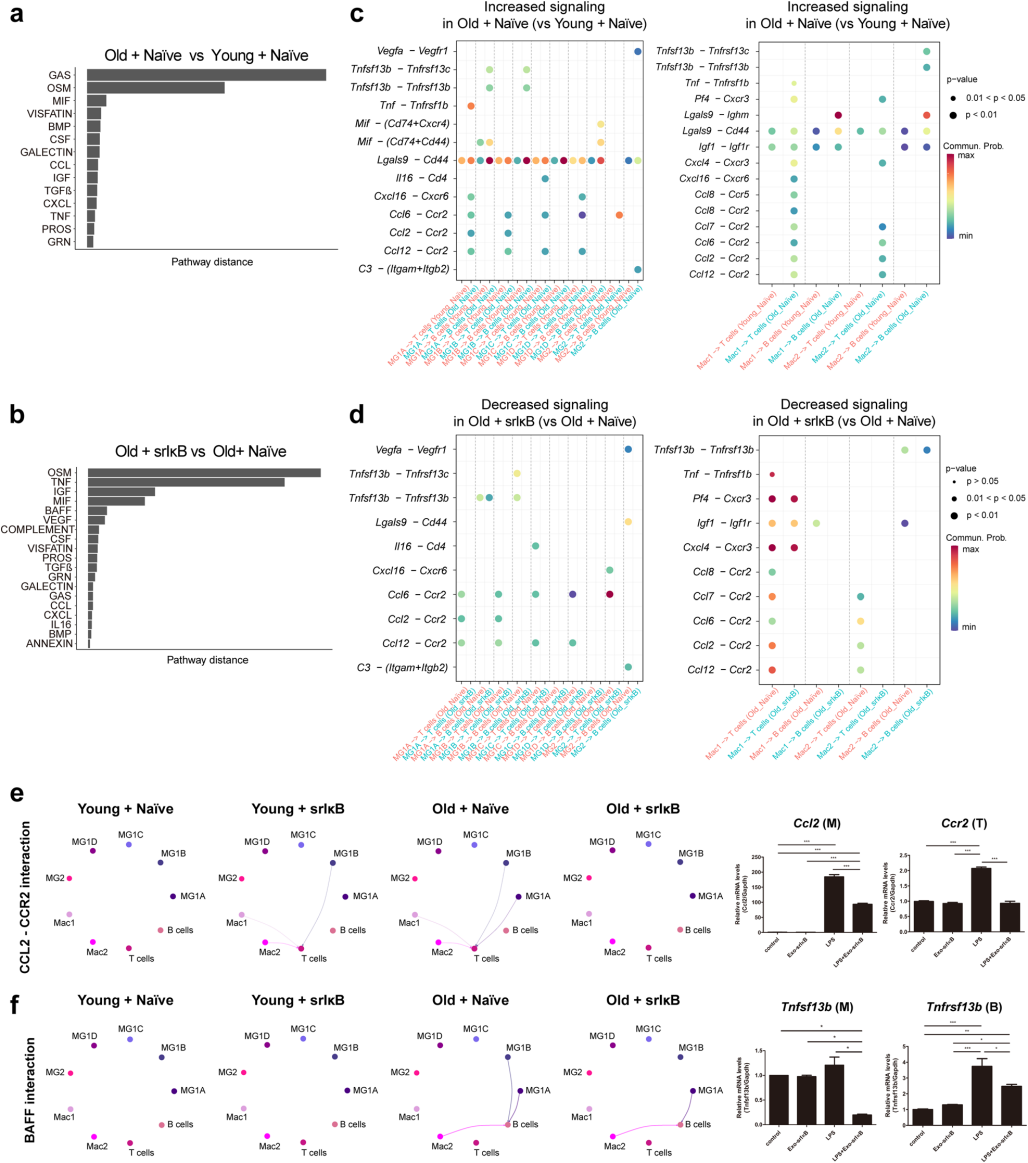
Analysis of cell‒cell communication among immune cells across the four distinct groups. **a**, **b** Signaling networks with a greater pathway distance in immune cells between Exo-Naïve-treated old vs. young mice (**a**) and Exo-srIκB-treated vs. Exo-Naïve-treated old mice (**b**). The pathway distance of specific signaling networks was based on their Euclidean distances in the shared two-dimensional space according to functional similarity. **c** Bubble plots illustrating ligand‒receptor pairs involved in interactions between microglia or macrophages and T cells or B cells that were significantly increased in old Exo-Naïve-treated mice compared with young Exo-Naïve-treated mice. The dot color and size denote the probabilities of communication from specific cell types to other cell types and the corresponding *p*-values, respectively. **d** Bubble plots illustrating ligand‒receptor pairs involved in interactions between microglia or macrophages and T cells or B cells that exhibited a significant decrease in old Exo-srIκB-treated mice compared with old Exo-Naïve-treated mice. The dot color and size denote the probabilities of communication from specific cell types to other cell types and the corresponding *p-*values, respectively. **e**, **f** (Left panels) Circle plots depict the interactions of selected signaling networks from microglia and macrophages to T and B cells across the four groups. Edge colors represent the source cell types of the interactions, and edge widths are proportional to the interaction strength. (Right panels) RAW264.7 cells (M) were stimulated with LPS (1 µg/ml) and Exo-srIκB (1 × 10^7^ particles) for 24 h, and then the conditioned media was used to treat EL4 (T) and FB2 (B) cells for 24 h. The expression of the *Ccl2, Ccr2, Tnfsf13b*, and *Tnfrsf13b* mRNAs was analyzed via qRT‒PCR and normalized to *Gapdh* expression (means ± SDs, *p-value < 0.05, **p-value < 0.01, and ***p-value < 0.001). Statistical significance was assessed using one-way ANOVA with Tukey’s multiple comparisons test.

The cell type composition and molecular signature analysis revealed that Exo-srIκB primarily targeted brain-infiltrating macrophages, followed by age-related interferon-responsive microglia. However, as T and B cells significantly infiltrated the brains of old mice and B-cell numbers were diminished after Exo-srIκB treatment (see Fig. 3b), we assessed the mechanism by which Exo-srIκB altered the communication between macrophages or microglia and T or B cells. To this end, we conducted a cell–cell communication analysis among immune cells from the scRNA-seq data using the R package CellChat^37^. First, a pathway distance analysis across the four conditions revealed that proinflammatory pathways (e.g., macrophage migration inhibitory factor (MIF), CCL, chemokine (C-X-C motif) ligand (CXCL), and tumor necrosis factor (TNF)) exhibited substantial differences between old and young Exo-Naïve-treated mice and between old Exo-srIκB-treated and Exo-Naïve-treated mice (Fig. 5a, b).

We further investigated the critical signaling pathways involved in the crosstalk between the various states of microglia or macrophages and T or B cells. Compared with those in young Exo-Naïve-treated mice, in old Exo-Naïve-treated mice, multiple ligand–receptor pair interactions were upregulated, particularly those involved in B-cell activating factor (BAFF) signaling (e.g., *Tnfsf13b–Tnfrsf13b*) from MG1A, MG1B, and Mac2 to B cells; CCR2-related signaling (e.g., *Ccl2–Ccr2, Ccl6–Ccr2, Ccl12–Ccr2*) from all states of microglia and macrophages to T cells; and CXCL16 signaling (e.g., *Cxcl16–Cxcr6*) from MG1A, MG1D, and Mac1 to T cells (Fig. 5c). BAFF signaling is a potent B-cell-activating pathway^57^. Furthermore, CCR2–expressing CD8^+^ T cells respond to chemokine action driven by microglia and infiltrate the brain^58,59^, and CXCL16–CXCR6 interactions contribute to the accumulation of CD8^+^ T cells in the brain^60,61^. Following Exo-srIκB treatment in old mice, the degree of interaction of multiple ligand–receptor pairs involved in CCR2-mediated signaling, especially the *Ccl2–Ccr2* pair, decreased from MG1A, MG1B, Mac1, and Mac2 to T cells (Fig. 5d, e (left panel)). Although the interactions of the *Cxcl16–Cxcr6* pair from microglia and macrophages to T cells increased with age, the overall communication network of *Cxcl16–Cxcr6* seemed even stronger following Exo-srIκB treatment, potentially explaining the sustained infiltration of T cells in old Exo-srIκB-treated mice, despite the suppression of CCR2-mediated signaling (Fig. 5d, Supplementary Fig. 12). With respect to BAFF signaling, after Exo-srIκB treatment, the interactions of the *Tnfsf13b–Tnfrsf13c* and *Tnfsf13b–Tnfrsf13b* pairs were downregulated in the crosstalk between MG1A, MG1B, and Mac2 and B cells (Fig. 5d, f (left panel)). Furthermore, the expression levels of *Ccr2* and *Tnfrsf13b* were elevated in T and B cells, respectively, in old Exo-Naïve-treated mice compared with young Exo-Naïve-treated mice and decreased after Exo-srIκB treatment (Supplementary Fig. 13a, b). Additionally, compared with those in young Exo-Naïve-treated mice, the expression levels of *Cxcl16* increased in microglia and macrophages from old Exo-Naïve-treated mice; however, compared with those in old Exo-Naïve-treated mice, the *Cxcl16* expression levels increased even more in MG1A, MG1B, MG1C, MG1D, Mac1, and Mac2 in old Exo-srIκB-treated mice (Supplementary Fig. 13c); again, this result partially explains the sustained infiltration of T cells in the brains of old Exo-srIκB-treated mice. We validated the results of the cell‒cell interactions, particularly between macrophages and T cells/B cells, from the scRNA-seq data by performing in vitro experiments using murine macrophages (RAW264.7) treated with Exo-srIκB + LPS, since LPS stimulation is commonly used to model neuroinflammation associated with aging and neurodegenerative diseases^6^. We collected the supernatants obtained from RAW264.7 macrophages treated with Exo-srIκB + LPS, treated T and B cells with these supernatants, and tested the expression levels of the *Ccl2–Ccr2* and *Tnfsf13b–Tnfrsf13b* pairs in macrophages–T cells and macrophages–B cells via qRT‒PCR. The expression levels of *Ccl2* and *Tnfsf13b* in RAW264.7 macrophages, *Ccr2* in T cells, and *Tnfrsf13b* in B cells increased upon LPS treatment but decreased significantly after Exo-srIκB treatment (*p* < 0.05) (Fig. 5e, f (right panel)). Overall, dynamic changes in interactions, particularly those associated with CCR2, CXCL16, and BAFF-related signaling, between innate immune cells in the brain and T and B cells suggest a possible mechanism underlying the infiltration of adaptive immune cells in the aged brain. Furthermore, the heterogeneous responses of cells participating in these dynamic interactions may explain the significant B-cell depletion in contrast to the sustained infiltration of T cells following Exo-srIκB treatment in the aged brain.

## Discussion

In the present study, we showed that NF-κB plays critical roles in the expression of numerous inflammation-associated genes in aged mouse brains. Exo-srIκB intervenes with age-related activated macrophages and interferon-responsive microglia in the brain, leading to a significant shift in the cell composition and interactions between microglia and macrophages with T and B cells. These findings suggest that Exo-srIκB is a potent therapeutic for treating age-related neuroinflammation.

Several studies have analyzed the profiles of inflammatory cells and changes in brain cells, including neurons and glial cells, in the aged brain using scRNA-seq or single-nucleus RNA sequencing^6,39,40^. Although some studies have successfully identified various types of mature neuronal cells from aged mouse brains via scRNA-seq^27^, single-cell dissociation of mammalian adult brains remains technically challenging. This difficulty is due to the highly interconnected nature of the tissue, which often results in the disruption of neuronal RNA integrity and inadequate capture of viable neuronal cells^27,38^. This poor identification of mature neuronal cells with scRNA-seq has been observed in a previous study^62^. The single-cell transcriptomic profiling method used in the present study focused on analyzing resident or infiltrating inflammatory cells and immune cells affected by Exo-srIκB in the aged brain. Our findings successfully revealed that Exo-srIκB treatment induced changes in the type, state, and composition of immune cells in the brain.

We found that aging altered the cell type and cell state composition of immune cells in the brain and altered the transcriptomic profile and differentiation of brain-infiltrating macrophages and microglia, which transitioned into an aging-related activated type that can produce several proinflammatory cytokines and chemokines, similar to findings from previous studies^6,39,40^. Our previous analyses of brain tissues from mice of various ages revealed that significant changes in the number of Iba-1-positive cells were clearly detected at 15 months^18^ and persisted from 18–22 months. We employed bulk RNA-seq (15 months old) identify factors that initiate age-related neuroinflammation at early stages of the aging process; thus, we were able to identify that one of the key transcription factors responsible for the increased levels of chemokines was NF-κB. We performed scRNA-seq at more advanced stages of aging (18–22 months old) to examine the effects of srIκB with greater accuracy. Our results indicate that scRNA-seq is a valuable tool for tracking and analyzing aging-associated molecular signatures and cellular changes and for verifying the effects of anti-inflammatory drugs, including exosomes.

Several studies have shown that the administration of extracellular vesicles or exosomes can alleviate inflammation and prevent aging^63–66^. Our results also revealed that cytokine/chemokine expression and inflammatory cell infiltration in the brains of old mice were lower in the group that was administered nonengineered exosomes alone (Exo-naïve) than in the group that did not receive the exosomes. However, unlike other substances, Exo-srIκB directly blocks the nuclear translocation of NF-κB without any additional mechanisms. Accordingly, Exo-srIκB inhibits the activities of specific NF-κB target genes or cells more selectively than nonengineered Exo-Naïve, making Exo-srIκB more effective for controlling inflammation than regular exosomes. In addition to age-related neuroinflammation, several studies have investigated the efficacy of Exo-srIκB. The administration of Exo-srIκB in a septic mouse model alleviated mortality and systemic inflammation^10^. Moreover, Exo-srIκB treatment during lipopolysaccharide-induced preterm birth prolonged gestation and reduced maternal inflammation^15^. Compared with the control, the systemic delivery of Exo-srIκB decreased the gene expression of proinflammatory cytokines and adhesion molecules in postischemic kidneys^16^. Furthermore, intraperitoneal Exo-srIκB administration alleviated mechanical allodynia and reduced the levels of cytokines and chemokines in a chronic postischemia pain model^14^. Previous studies have focused only on the effects of Exo-srIκB on relatively acute inflammation; however, this study also revealed their effects on chronic inflammation caused by aging and various other factors.

Although the SCENIC analysis did not reveal significant changes in the expression of NF-κB target genes, we observed a significant reduction in the expression levels of NF-κB target proteins, as shown by the results of the cytokine/chemokine array. Since we analyzed the brains of the mice 1 day after three consecutive days of Exo-srIκB administration, this time point may reflect changes in NF-κB-mediated protein expression and immediate early immune responses rather than transcriptional changes in NF-κB target genes. Moreover, we confirmed that Exo-srIκB led to a notable shift in the composition of microglia and macrophages, particularly through a significant reduction in age-related interferon-responsive MG2 and activated Mac2, both of which are governed by proinflammatory TFs. These results suggest that Exo-srIκB significantly alters the immune microenvironment of the brain by modulating NF-κB signaling across various biological pathways in immune cells, which are tightly regulated through autoregulatory feedback loops^67,68^.

**Fig. 6:**
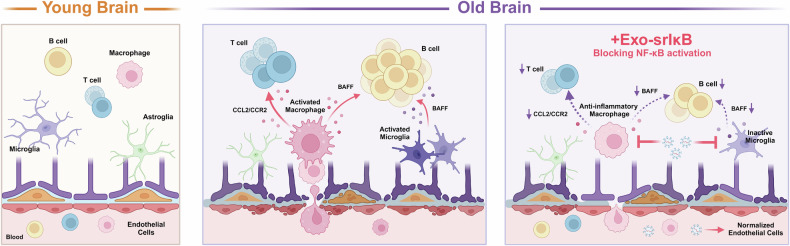
Schematic model depicting the role of Exo-srIκB in age-related neuroinflammation.

In old Exo-srIκB-treated mice, the expression levels of 35 cytokines and chemokines (among the 79 whose levels were increased), including IL-1α, IL-11, and IL-15, were reduced compared with those in old Exo-Naïve-treated mice. Although the levels of 44 cytokines and chemokines were not significantly affected by Exo-srIκB in aged mice, Exo-srIκB treatment significantly reduced the number of infiltrating B cells in the brains of old mice and altered critical signaling pathways involved in the crosstalk between microglia or macrophages and T cells or B cells, such as CCR2 and BAFF. Our single-cell transcriptomic profiling data from the brains of mice treated with Exo-srIκB and Exo-Naïve strongly suggest that brain-infiltrating macrophages and interferon-responsive microglia are the main targets of Exo-srIκB, as shown by the cell type composition and molecular signature identification analyses. Moreover, in the analysis of transdifferentiation based on the trajectories of macrophage states across the four groups (young/old and Exo-Naïve/Exo-srIκB), we found that Exo-srIκB successfully altered the transcriptomic profiles and differentiation of brain-infiltrating macrophages, significantly shifting them from an age-related activated type to a less activated type. Collectively, our results demonstrate that Exo-srIκB affects cytokines and chemokines secreted by macrophages/microglia and factors associated with T and B cells and induces integrated changes between immune cells in the aged brain; thus, Exo-srIκB may serve as a potent therapeutic agent against pathological age-related inflammatory processes, especially those that target macrophages and microglia (Fig. 6).

Although many NF-κB inhibitors have been developed and proposed, IκB protein antagonists prepared by specific activation mechanisms may act more selectively than pharmacological antagonists^69^. Here, we utilized engineered exosomes loaded with a nondegradable form of IκB and showed that they could induce phenotypic changes in macrophages and microglia, which in turn prompted changes in T and B cells, potentially leading to beneficial effects on age-related neuroinflammation. In terms of efficacy and safety, an optogenetically engineered exosome system (EXPLOR technology), which specifically loads the functional dominant active form of the IκB protein into exosomes, can potentially minimize off-target side effects compared with the use of other small chemical-based NF-κB inhibitors^13^. However, inhibiting canonical NF-κB signaling may activate or affect the noncanonical NF-κB signaling pathway, as reported in previous studies^70^. Potential on-target side effects include immunosuppression or cell death due to excessive inhibition of NF-κB activity; however, in the current preclinical animal experiments, these effects were not observed even after treatment with high concentrations of Exo-srIκB. The inhibition of NF-κB activity was achieved via proteins rather than small chemicals, contributing to high specificity and suggesting that off-target effects are expected to be rare.

In the present study, we decided to inject the exosomes three times instead of once because a large number of systematically delivered exosomes were captured in the liver^71^. We observed biological changes in Iba-1-positive cells and alterations in the interactions between microglia and macrophages and T and B cells through an scRNA-seq analysis following short-term Exo-srIκB treatment in aged mice. However, histological analysis did not reveal significant changes in the number or morphology of Iba-1-positive cells. Since morphological changes may not be detectable with short-term administration, we plan to investigate histological changes, along with cognitive and behavioral improvements, in aging models through long-term treatment with Exo-srIκB in future studies. Although these aspects were not tested here, we believe that observing the effects of Exo-srIκB on a model of age-related neuroinflammation under the conditions assessed in this study is very encouraging.

To the best of our knowledge, our study is the first to focus on the application of Exo-srIκB to ameliorate neuroinflammation using scRNA-seq, indicating the possibility of controlling low-grade chronic inflammation in the aged brain by delivering therapeutic proteins involved in NF-κB inactivation via exosomes, which are powerful biocompatible nanoparticles. More work remains to be performed in the field of exosome-based therapeutics, including the evaluation of various surface modifications for active targeting and quality control. Furthermore, the single-cell dissociation and sample preparation protocols for scRNA-seq applied herein were insufficient to capture mature, highly diverse neuronal cells, representing a limitation of our study. Nevertheless, our findings will substantially contribute to the further development of next-generation drug delivery systems for the treatment of age-related neuroinflammation and neurodegenerative disorders.

## Supplementary information

Supplementary information

## Data Availability

The raw sequence data presented in this study have been deposited in the Korea Sequence Read Archive (KRA) at the Korea Bioinformation Center, Korea Research Institute of Bioscience and Biotechnology (accession number: KAP240788), and are publicly accessible at https://kbds.re.kr/KRA. Other data are available from the corresponding author upon reasonable request.
