# Supplementary material for: Exosome-based targeted delivery of NF-κB ameliorates age-related neuroinflammation in the aged mouse brain
Source: Exp Mol Med. 2025 Jan 20;57(1):235–48. doi: 10.1038/s12276-024-01388-8 (PMC11799301; doi:10.1038/s12276-024-01388-8)
Supplement: Supplementary file 1 — Supplementary information [file 12276_2024_1388_MOESM1_ESM.pdf]

Supplementary information

Exosome-based targeted delivery of NF-κB ameliorates age-related neuroinflammation in the aged mouse brain

Chae-Jeong Lee[1,#], Seung Hyun Jang[2,#], Jiwoo Lim[1], Hyunju Park[1], So-Hee Ahn[3], Seon Young Park[3], Hyangmi Seo[3], Soo-Jin Song[4], Jung A Shin[4], Chulhee Choi[3,*], Heon Yung Gee[2,*], Youn-Hee Choi[1,*]

[1]Department of Physiology, Inflammation-Cancer Microenvironment Research Center, Ewha Womans University College of Medicine, Seoul 07804, Republic of Korea.

[2]Department of Pharmacology, Brain Korea 21 PLUS Project for Medical Sciences, Yonsei University College of Medicine, Seoul 03722, Republic of Korea.

[3]ILIAS Biologics Inc., Daejeon 34014, Republic of Korea.

[4]Department of Anatomy, Ewha Womans University College of Medicine, Seoul 07804, Republic of Korea.

[#]These authors contributed equally to this work.

[*]Author to whom correspondence should be addressed.

*Corresponding author

E-mail: yc@ewha.ac.kr (Y.-H.C.), hygee@yuhs.ac (H.Y.G.), cchoi@iliasbio.com (C.C.)

This file contains Supplementary Methods, Supplementary Figures 1-13, and Supplementary Tables 1.

Supplementary Methods

Immunohistochemistry

Paraffin-embedded tissue blocks were sectioned into 3-μm-thick slices for immunohistochemistry analysis. The sections were deparaffinized and then incubated in endogenous peroxidase and protein-blocking solution at room temperature. The primary antibody was used a rabbit polyclonal antibody targeting ionized calcium-binding adapter molecule 1 (Iba-1) (FUJIFILM Wako Chemicals, Japan), diluted in 0.01 M PBS. The sections were incubated with this primary antibody overnight at 4°C. The following day, after washing, the sections were incubated with horseradish peroxidase (HRP)-conjugated goat anti-rabbit IgG (Jackson ImmunoResearch Laboratories, USA) at room temperature for 1.5 h. After another washing step, the sections were stained with the DAB+ chromogenic substrate (Agilent, CA), followed by dehydration and mounting with Permount (Thermo Fisher Scientific, MA). Images of the stained sections were obtained using either an Olympus VS200 slide scanner or a BX-50 light microscope (Olympus, Japan). These images were analyzed using ImageJ software (NIH, version 1.53). To measure the Iba-1-positive cell numbers, 10 to 15 images were taken from each slide, focusing on specific brain regions of each group within a 500 × 600 μm field. Two independent operators counted the numbers of DAB-stained Iba-1-positive cells in the brain.

Western blot analysis

For the preparation of brain lysates, the fresh cerebral cortex of the mouse brain tissues was lysed using a sonicator (BIORUPTOR®II Type6; Cosmo Bio, USA) in 200 μL of ice-cold radioimmunoprecipitation assay (RIPA) lysis buffer with the Xpert protease inhibitor cocktail (GenDEPOT, USA). For the preparation of cell and exosome lysates, the exosome-producing cells were lysed with RIPA buffer (Thermo Fisher Scientific, cat. no. 89900) containing Halt™

Protease and Phosphatase Inhibitor Cocktail (100×) (Thermo Fisher Scientific, cat. no. 1861281); the exosomes were lysed with 4× Laemmli sample buffer (Bio-RAD, cat. no. 161-0747). The total proteins were separated on 12% sodium dodecyl sulfate-polyacrylamide gels and transferred onto polyvinylidene fluoride or nitrocellulose membranes. The resulting blots were blocked with 5% skim milk in tris-buffered saline (TBS) or TBS-T (containing 0.1% Tween-20) for 1 h at room temperature and incubated overnight at 4°C with the primary antibodies (anti-IκBα (Cell Signaling Technology, cat. no. 4814), anti-NF-κB p65 (Cell Signaling Technology, cat. no. 8242), and anti-tubulin (Sigma-Aldrich, cat. no. T5168)) and other exosome- and cell organelle-specific markers. After washing with TBS-T, the membranes were incubated with specific secondary antibodies for 1 h at room temperature and washed again in TBS-T. The protein bands were visualized using Clarity Max ECL Western Blotting Substrates (Bio-Rad, CA, USA) or Amersham ECL Western Blotting Detection Reagent (Cytiva, UK) and imaged using a ChemiDoc imager (Bio-Rad, CA, USA) or ImageQuant™ LAS 4000 (GE Healthcare, UK). To confirm the major positive and negative markers of exosomes, antibodies targeting the following proteins were used: srIκB, CRY2 (Abclon, customized antibody), CD9 (SBI, cat no. EXOAB-CD9A-1), CD81 (SBI, cat no. EXOAB-CD81A-1), TSG101 (Abcam, cat no. ab228013), GM130 (Abcam, cat no. ab52649), GAPDH (Santa Cruz Biotechnology, cat no. sc-47724).

RNA extraction and bulk RNA sequencing

Total RNA was extracted from the cerebral cortex of young (3 months old, n = 6 (4 female, 2 male)) and old mice (15 months old, n = 7 (3 female, 4 male)) using a Qiagen RNA extraction kit (Qiagen, cat no. 79306; Valencia, CA, USA); clean-up was performed using the RNeasy mini kit (Qiagen, cat no. 74106; Valencia, CA, USA), following the manufacturer's instructions. RNA sequencing was performed by Macrogen (Seoul, Korea). Total RNA concentration was measured using Quant-IT RiboGreen (Invitrogen, cat no. R11490). Samples were analyzed using the TapeStation RNA ScreenTape (Agilent, cat no. 5067-5576) to assess the integrity

of the total RNA. High-quality RNA samples with RNA integrity number (RIN) values higher than 7.0 were subjected to RNA library construction. Libraries were constructed using an Illumina TruSeq Stranded mRNA Library Sample Prep Kit (Illumina, cat no. RS-122-2101; San Diego, CA, USA); the enriched libraries were sequenced on an Illumina NovaSeq sequencing system (Illumina, Inc.; San Deigo, Ca, USA). The CLC Genomics Workbench 9.5.3 software (Qiagen, Germany) was used to map the reads to the mouse genome (GRCm39/mm39).

Tissue dissociation and preparation of single-cell suspensions

The fresh specimens were immediately stored in the tissue storage solution (Miltenyi Biotech, cat no. 130-100-008) at 4°C. Before dissociation, the tissues were washed with PBS and minced into 1–2-mm pieces. The tissue pieces were then dissociated using the Neural Tissue Dissociation Kit (P) (Miltenyi Biotech, cat no. 130-092-628) and gentleMACS dissociator (Miltenyi Biotech), following the manufacturer's instructions. Following dissociation, the cell suspensions were sequentially passed through 70- and 30-μm strainers to separate the cells from the debris and undigested tissue chunks. The cells were then washed two times with cold Ca^{2+} and Mg^{2+}-free 0.04% BSA/PBS; the resulting suspensions were centrifuged at 300 *g* for 5 min at 4°C. Finally, the samples were gently resuspended in stain buffer (BD Biosciences, cat no. 554656), and then counted using a LUNA-FX7™ Automated Fluorescence Cell Counter (Logos Biosystems) using acridine orange (AO) and propidium iodide (PI) staining (Logos Biosystems, cat no. F23001).

Multiplexing, library construction, and single-cell RNA sequencing

To multiplex the samples, cells from different conditions were tagged with antibody-polyadenylated DNA barcodes for mouse cells (BD Biosciences, cat no. 626545). The cells were stained with multiplexing antibody for 20 min at room temperature and then washed three times using stain buffer (BD Biosciences, cat no. 554656). After the final wash, the cells were resuspended in cold sample buffer (BD Biosciences, cat no. 664887) and counted using a

LUNA-FX7™ Automated Fluorescence Cell Counter (logos biosystems); then 7,000 target cells from each condition were pooled. Single-cell capture was performed using the BD Rhapsody Express instrument according to the manufacturer's instructions (BD Bioscience). Pooled cells from each group in a cold sample buffer were loaded into the BD Rhapsody cartridge (BD Biosciences, cat no. 666262). After cell separation, cell barcode-magnetic beads were inserted into the cartridge. Then, the cells were lysed and the mRNA capture beads were retrieved. Subsequently, the mRNA capture beads were subjected to cDNA synthesis and Exonuclease I treatment using the BD Rhapsody cDNA kit (BD Bioscience, cat no. 633773). According to the 'mRNA Whole Transcriptome Analysis (WTA) and Sample Tag Library Preparation' protocol (BD Bioscience), scRNA-seq libraries were constructed using the BD Rhapsody WTA amplification kit (BD Bioscience, cat no. 633801). For the WTA library, the cDNA underwent random priming and extension, amplification, and index PCR. For the sample tag library, the cDNA underwent nested PCR (PCR 1 and PCR 2) and index PCR. The purified WTA and sample tag libraries were quantified using qPCR performed following the qPCR Quantification Protocol Guide (KAPA) and subjected to quality assessment using the Agilent Technologies 4200 TapeStation instrument (Agilent technologies). The libraries were then pooled and sequenced on the HiSeq platform (Illumina, Inc.; San Deigo, Ca, USA), generating 150 bp paired-end reads. The BD Rhapsody WTA analysis pipeline (version 1.11.1) was used for sample de-multiplexing, barcode processing, and single-cell gene- unique molecular identifier (UMI) counting against the GRCm39 mouse reference genome with default parameters.

Scoring the activity of a specific gene set in each cell

The following genes were used for calculating the microglia interferon response scores: *Stat1, Ifit3, Ifitm3, Usp18, Ifi27l2a, Rtp4, Irf7, Isg15, Oasl2*[1,2]. The following genes were used for calculating the microglia activation scores: *B2m, Trem2, Ccl2, Apoe, Axl, Itgax, Cd9, C1qa, C1qc, Lyz2, Ctss*[3]. The macrophage activation scores were calculated using the following

genes: *Cd86, Cd40, Cd14, Il1b, Il18, Tnf, Mmp9, Itgax, Ccl2, Ccl3, Ccl4, Ccr2, Tlr2, Tlr4*[1,3-5]. The following genes were used for calculating the oligodendrocyte interferon response scores: *Stat1, Ifi27l2a, B2m, Usp18, and H2.K1*[2]. The genes used for calculating the demyelination-associated and myelination process-associated scores are provided in Supplementary Table 1[6]. The following genes were used for calculating the astrocyte activation scores: *C4b, C3, Serpina3n, Cxcl10, Gfap, Vim, Il18, Hif3a*[7-9]. For the calculation of T cell and B cell activation scores, genes belonging to the following gene sets were used: GOBP_B_CELL_ACTIVATION_INVOLVED_IN_IMMUNE_RESPONSE (GO:0002312) and GOBP_T_CELL_ACTIVATION_INVOLVED_IN_IMMUNE_RESPONSE (GO:0002286).

Quantitative real-time polymerase chain reaction (qRT-PCR)

Total RNA was extracted from cells using the easy-BLUE™ Total RNA Extraction Kit (iNtRON Biotechnology, cat no. 1706). cDNAs were synthesized from 1 μg of total RNA with ReverTra AceTM qPCR RT Master Mix (Toyobo, Japan, cat no. FSQ-201) using a thermal cycler (Bio-Rad, CA, USA) according to the manufacturer's protocol. Quantitative real-time PCR (qRT-PCR) was performed on an ABI StepOnePlus™ Real-Time PCR System instrument (Applied Biosystems, CA, USA) with 10 μL of SYBR® Green Real-time PCR Master Mix (Toyobo, cat no. QPK-201) per well. Melting curve analysis was performed for SYBR Green-based amplification by plotting the fluorescence intensity using the StepOne™ software (version 2.3). The expression levels of Gapdh (reference) were used to normalize the levels of the mRNAs of the target genes. The relative expression levels of the target genes were calculated using the 2−ΔΔCT method. The primer sequences were as follows: Ccl2, forward 5′-GCTACAAGAGGATCACCAGCAG-3′, reverse 5′-GTCTGGACCCATTCCTTCTTGG-3′; Ccr2, forward 5′-GCTGTGTTTGCCTCTCTACCAG-3′, reverse 5′-CAAGTAGAGGCAGGATCAGGCT-3′; Tnfsf13b, forward 5′-CTACCGAGGTTCAGCAACACCA-3′, reverse 5′-GAAAGCGCGTCTGTTCCTGTGG-3′; Tnfrsf13b, forward 5′-GGTCAGACAACTCAGGAAGGCA-3′, reverse 5′-

CCAAGAAACAGCAGAAGATGGCG-3′; Gapdh, forward 5′-CATCACTGCCACCCAGAAGACTG-3′, reverse 5′-ATGCCAGTGAGCTTCCCGTTCAG-3′.

References

1. Hammond, T. R. *et al.* Single-cell RNA sequencing of microglia throughout the mouse lifespan and in the injured brain reveals complex cell-state changes. *Immunity.* **50**, 253-271.e256 (2019).
2. Kaya, T. *et al.* CD8(+) T cells induce interferon-responsive oligodendrocytes and microglia in white matter aging. *Nat. Neurosci.* **25**, 1446-1457 (2022).
3. Allen, W. E., Blosser, T. R., Sullivan, Z. A., Dulac, C. & Zhuang, X. Molecular and spatial signatures of mouse brain aging at single-cell resolution. *Cell* **186**, 194-208.e118 (2023).
4. Jurga, A. M., Paleczna, M. & Kuter, K. Z. Overview of general and discriminating markers of differential microglia phenotypes. *Front. Cell Neurosci.* **14**, 198 (2020).
5. Zhang, C., Yang, M. & Ericsson, A. C. Function of macrophages in disease: current understanding on molecular mechanisms. *Front. Immunol.* **12**, 620510 (2021).
6. Hou, J. *et al.* Transcriptomic atlas and interaction networks of brain cells in mouse CNS demyelination and remyelination. *Cell Rep.* **42**, 112293 (2023).
7. Clarke, L. E. *et al.* Normal aging induces A1-like astrocyte reactivity. *Proc. Natl. Acad. Sci. USA* **115**, e1896-1905 (2018).
8. Liddelow, S. A. *et al.* Neurotoxic reactive astrocytes are induced by activated microglia. *Nature* **541**, 481-487 (2017).
9. Zamanian, J. L. *et al.* Genomic analysis of reactive astrogliosis. *J. Neurosci.* **32**, 6391-6410 (2012).

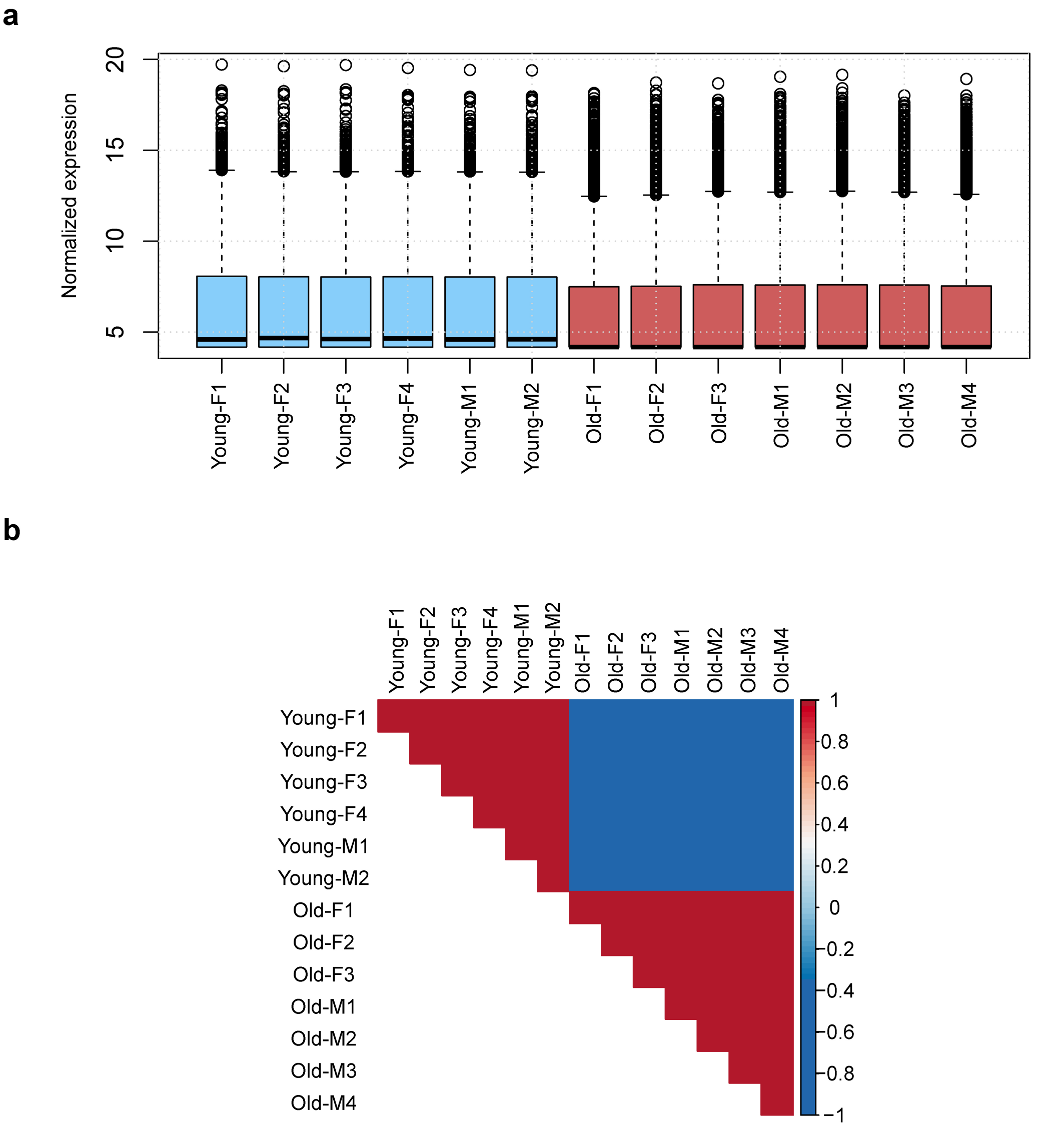

Supplementary Fig. 1. Quality evaluation of gene expression in bulk RNA-seq data.

a Box plots showing the distribution of normalized expression in each sample. Normalization was performed using the variance stabilizing transformation method provided by the R package DESeq2 (version 1.38.3). **b** Spearman correlation plots of normalized expression among different samples. Correlation coefficients between specific pairs of samples are colored, with red indicating positive correlation and blue indicating negative correlation.

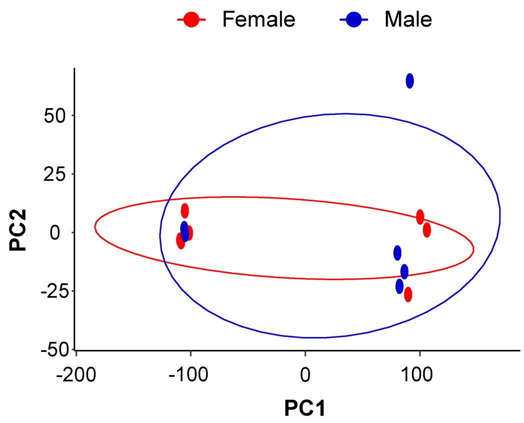

Supplementary Fig. 2. Principal component analysis of bulk RNA-seq data grouped by sex.

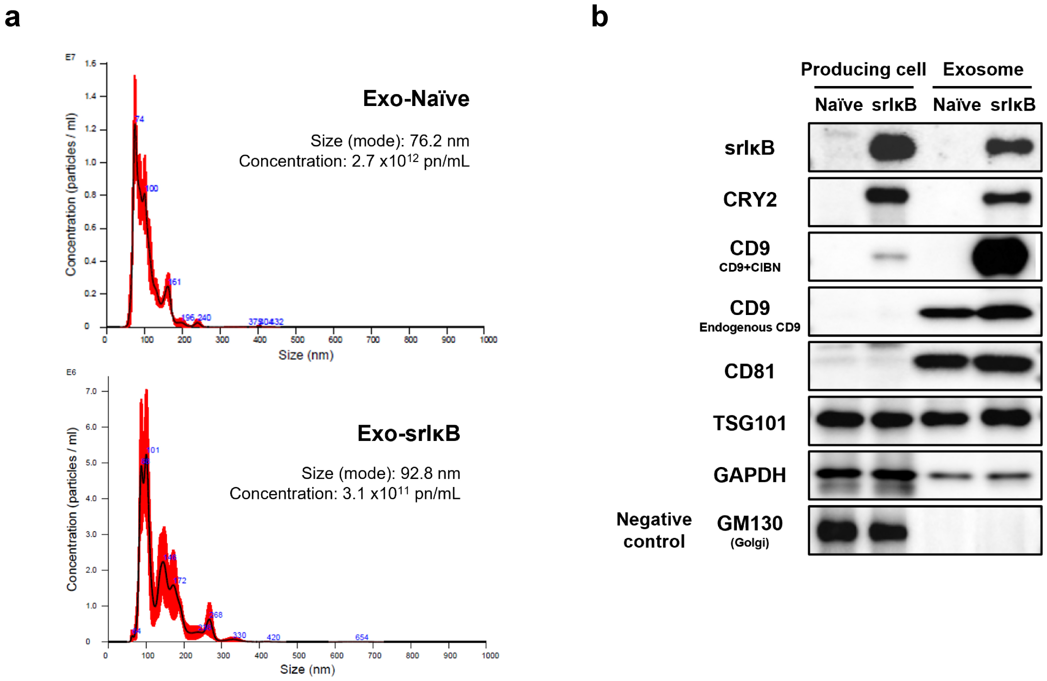

Supplementary Fig. 3. Characterization of engineered exosomes Exo-srIκB.

a Representative panels of nanoparticle tracking analysis (NTA) demonstrating concentration and size distribution of the Exo-Naïve (top) and Exo-srIkB (bottom) exosomes by a Nanosight (NS300). **b** Immunoblotting experiment of producing cells (Expi293F cells) and exosomes to analyze the expression of target protein (srIκB, CRY2, CD9), exosome positive markers (endogenous CD9, CD81, TSG101, GAPDH), and exosome negative markers (cell organelle markers; GM130). Exo-Naïve was used as a negative control of Exo-srIκB.

a

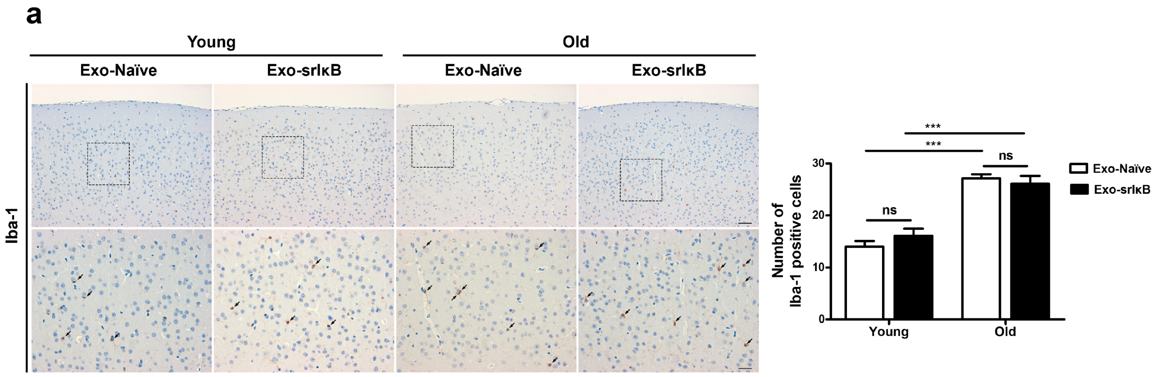

b

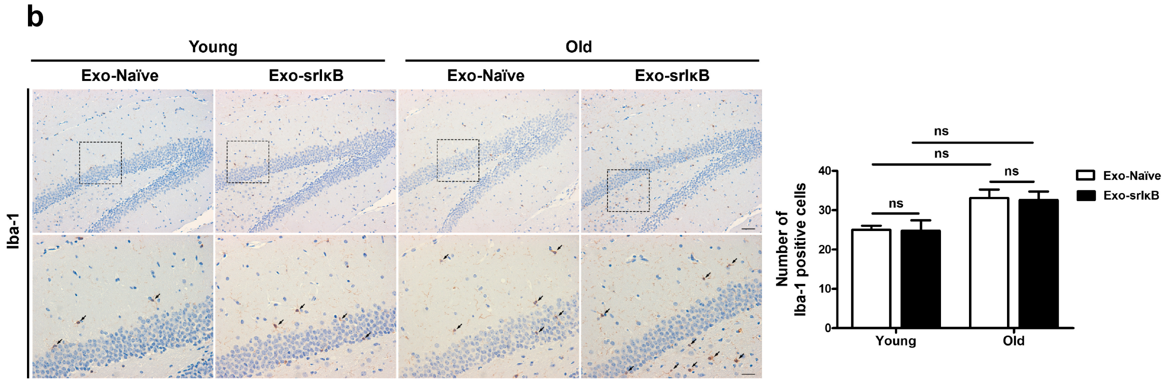

Supplementary Fig. 4. Immunohistochemical staining of Iba-1 in the mouse cortex and hippocampus across four groups.

a, b Immunohistochemical staining for Iba-1 in the cerebral cortex (**a**) and hippocampus (**b**) of young (3 months old) and old (19–22 months old) mice treated with Exo-Naïve or Exo-srIκB. The number of Iba-1-positive cells (arrows) was counted in the 0.3 mm^2 area in the cerebral cortex and hippocampus of four groups. The bars represent SD. ns: not significant; ****p*-value < 0.001. Scale bar = 200 μm in up, 50 μm in down.

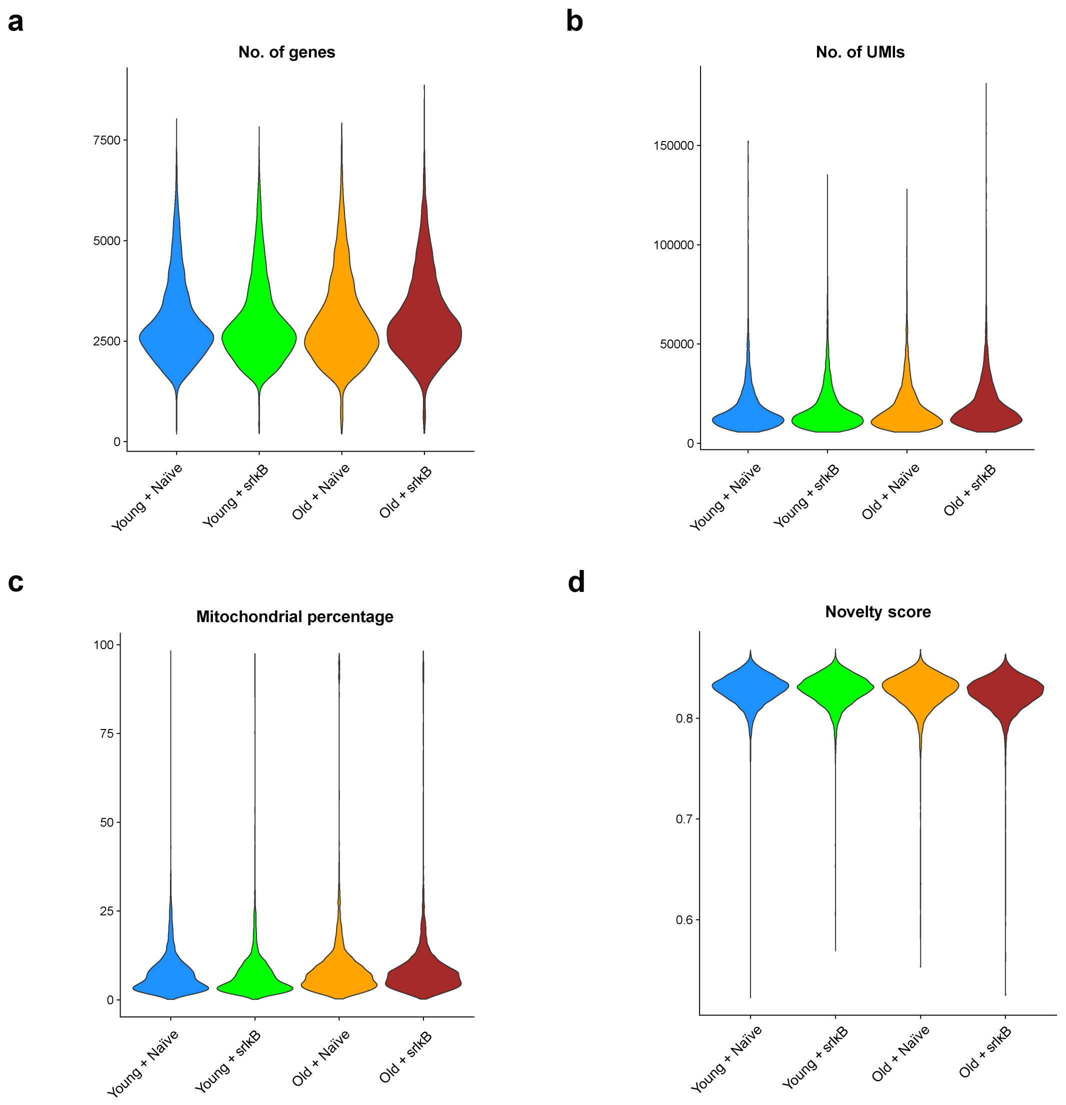

Supplementary Fig. 5. Violin plots displaying the quality control metrics of the single-cell RNA-sequencing data.

a The number of detected genes per sample. **b** The number of unique molecular identifiers (UMI) counts per sample. **c** The percentage of mitochondrial UMIs per sample. **d** The distribution of the novelty score is defined as the log10 of the number of detected genes divided by the log10 of the number of UMI counts.

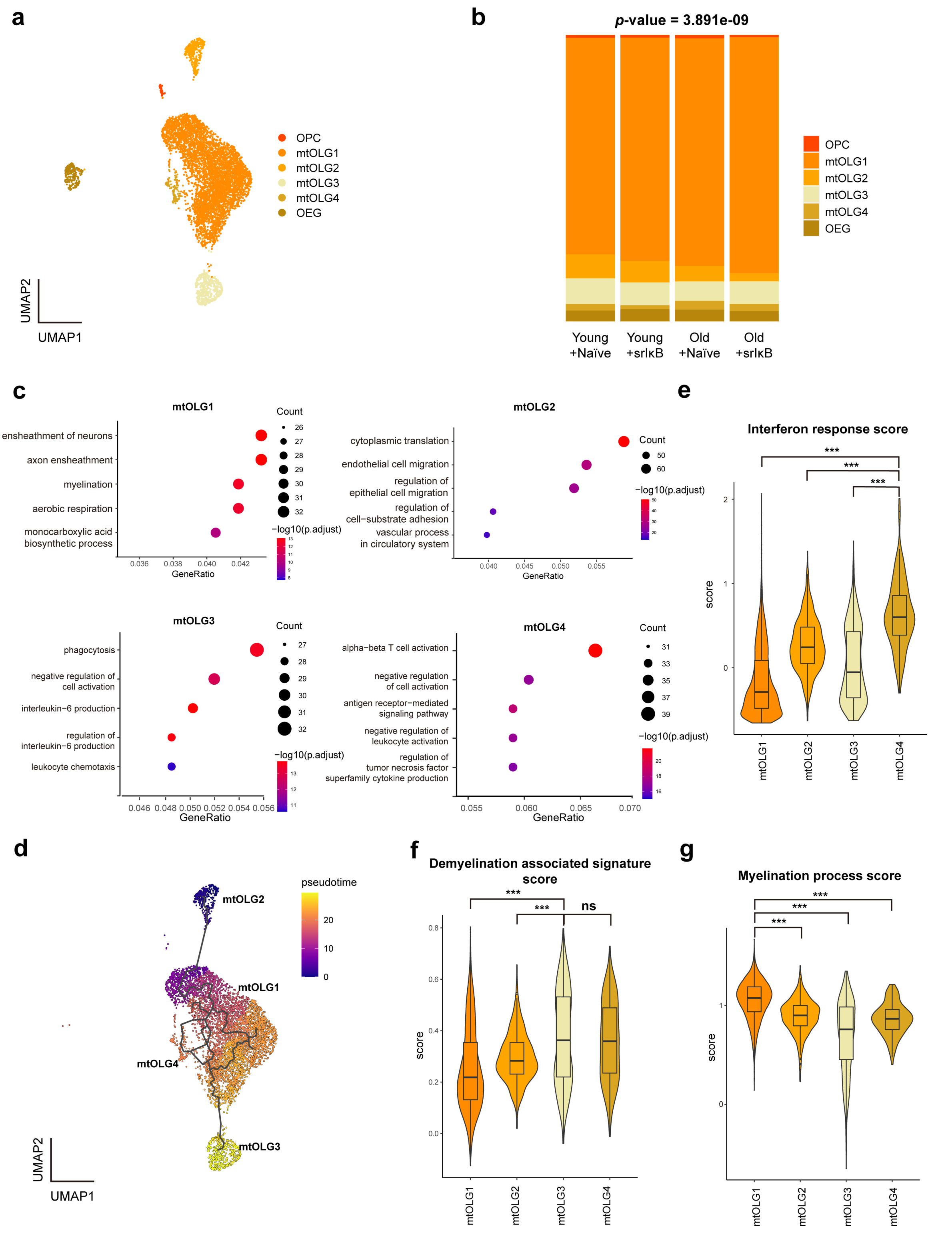

Supplementary Fig. 6. Comparison of cell-type composition and the molecular signature within the oligodendrocyte lineage across four groups.

a UMAP plot of the oligodendrocyte lineage cells (*n* = 6,412). **b** Proportion of cell types among the oligodendrocyte lineage across four different groups. A chi-square test was conducted to determine *p*-values**.** **c** Gene Ontology (GO) terms enriched among marker genes identified in each oligodendrocyte cluster. The dot color and size represent the *p*-value and gene ratio, respectively. The top 5 GO terms, according to the gene ratio, with BH-adjusted *p*-value < 0.05, are listed. **d** UMAP plot with trajectories of mature oligodendrocytes, colored by pseudotime. **e-g** Violin plots showing the interferon response (**e**), demyelination-associated (**f**), or myelination process-associated (**g**) scores among different cell types of mtOLG. *** indicates an adjusted *p*-value < 0.001, and ns indicates not statistically significant (one-way ANOVA test with Bonferroni multiple comparison correction) between two compared groups.

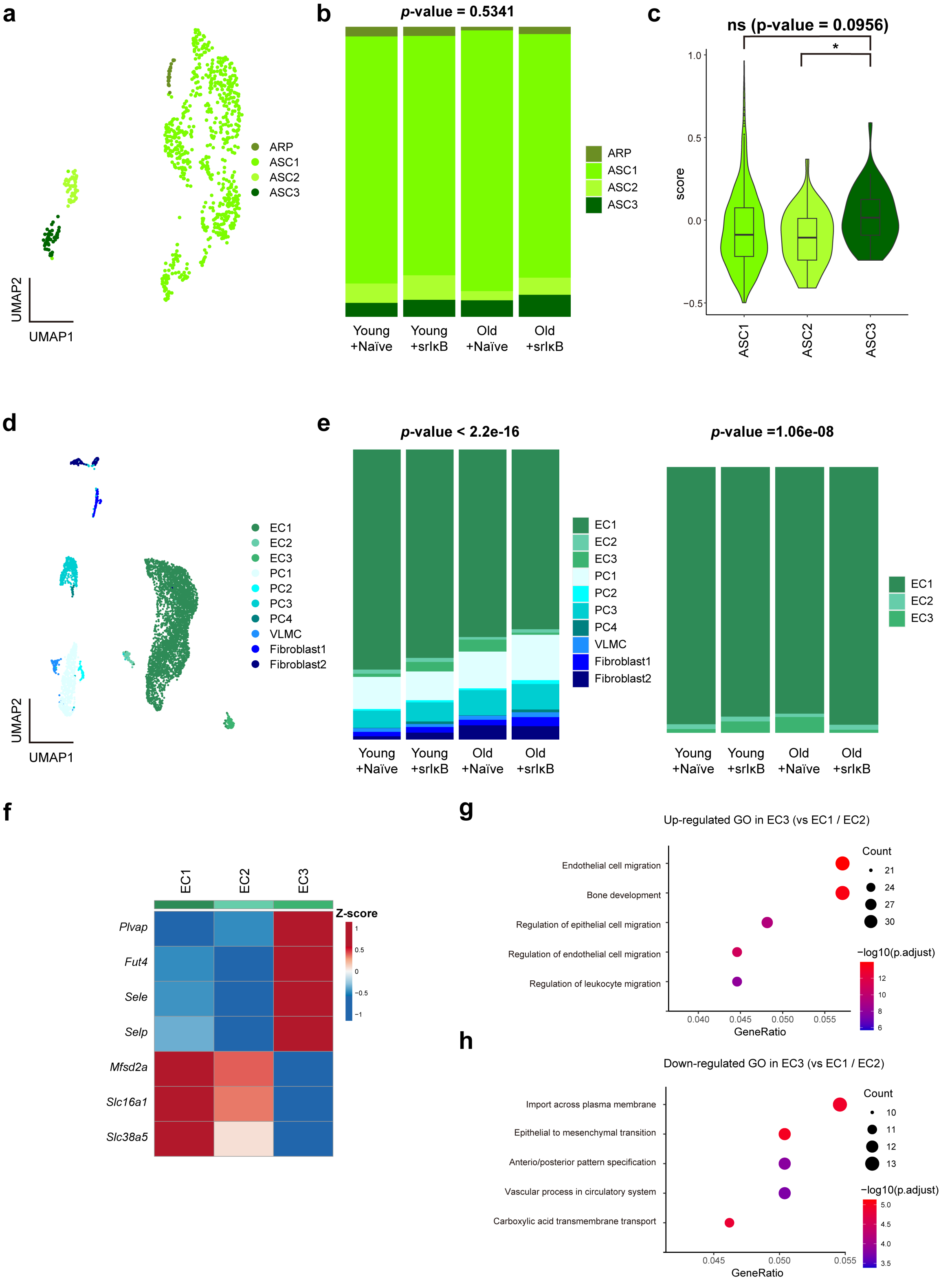

Supplementary Fig. 7. Comparison of cell-type composition and the molecular signature within the astrocyte lineage and vasculature cells across four groups.

a UMAP plot of the astrocyte lineage cells (*n* = 796). **b** Proportion of cell types among the astrocyte lineage across four different groups. A chi-square test was conducted to determine *p*-values. **c** Violin plots showing the astrocyte activation score among different cell types of mature astrocytes. ns: not significant; **p*-value < 0.05, one-way ANOVA test with Bonferroni multiple comparison correction. **d** UMAP plot of the vasculature cells (*n* = 5,392). **e** Proportion of cell types among all types of vasculature cells (left) or endothelial cells (right) across four different groups. A chi-square test was conducted to determine *p*-values. **f** Heatmap showing the Z-scored, normalized expression levels of representative differentially expressed genes (DEGs) in EC3 compared with EC1 and EC2. **g-h** Gene Ontology (GO) terms enriched among significantly upregulated (**g**) or downregulated (**h**) DEGs in EC3 compared with EC1 and EC2. The dot color and size represent the *p*-value and gene ratio, respectively. The top 5 GO terms, according to the gene ratio, with BH-adjusted *p*-value < 0.05, are listed.

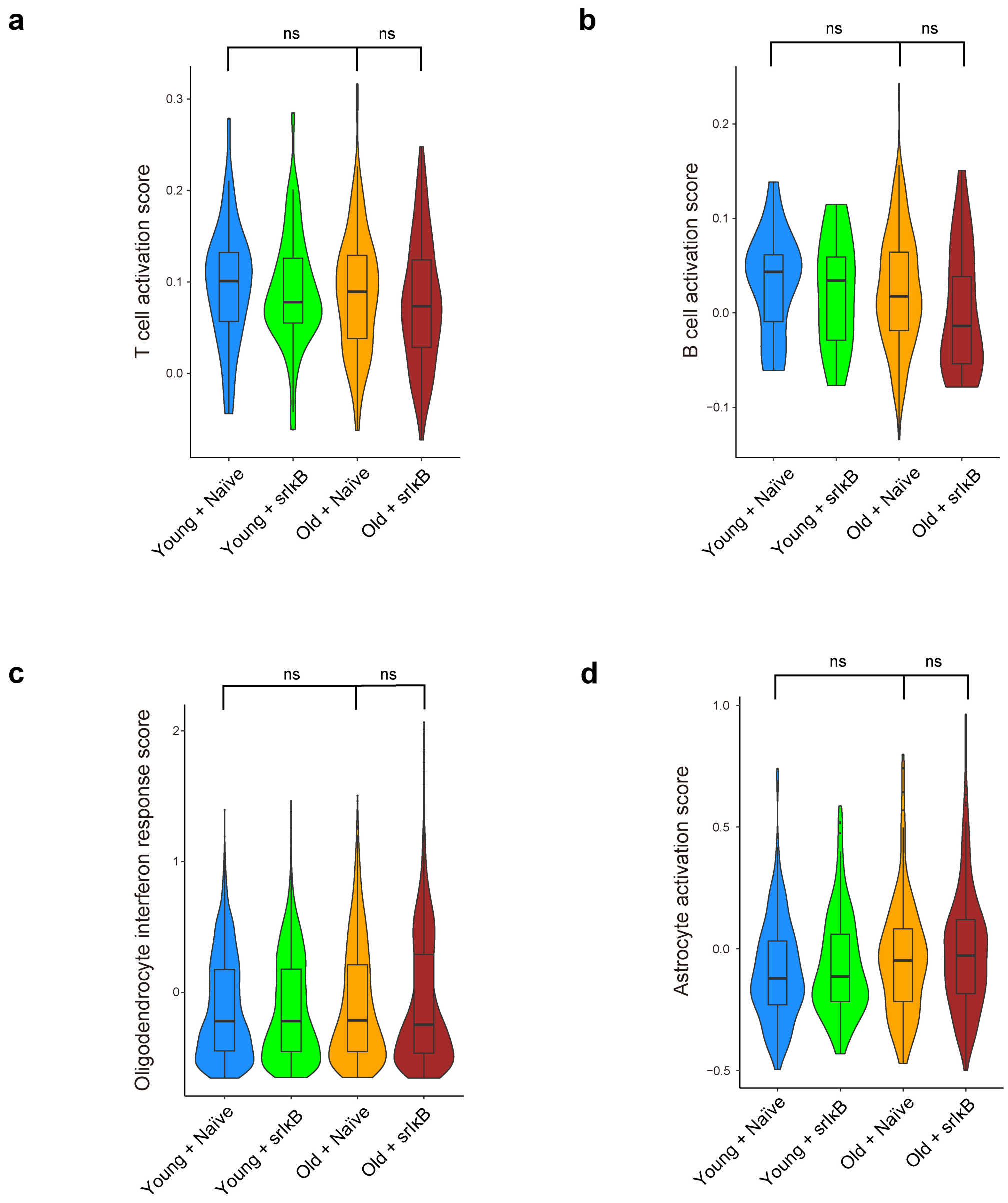

Supplementary Fig. 8. Violin plots presenting the scores of selected molecular signatures across four groups.

a-d Violin plots presenting the scores of selected molecular signatures across four groups within T cells (**a**), B cells (**b**), oligodendrocytes (**c**), and astrocytes (**d**). ns: not significant, one-way ANOVA test with Bonferroni multiple comparison correction.

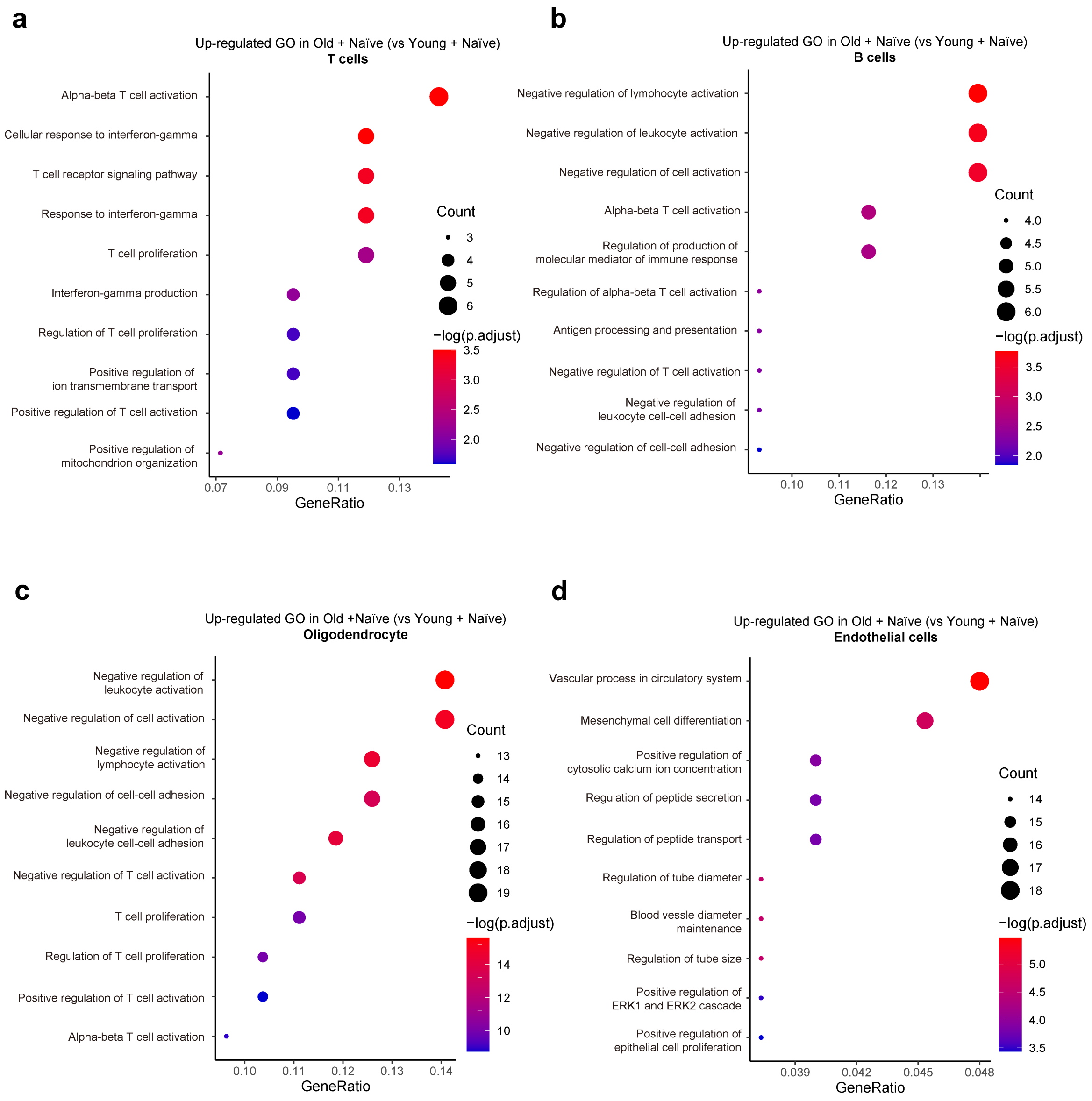

Supplementary Fig. 9. GO terms enriched among significantly upregulated DEGs.

a-d GO terms enriched among significantly upregulated DEGs in T cells (**a**), B cells (**b**), oligodendrocyte (**c**), and endothelial cells (**d**) in Exo-Naïve-treated old mice compared with those in Exo-Naïve-treated young mice. The dot color and size represent the *p*-value and gene ratio, respectively. The top 10 GO terms, according to the gene ratio, with BH-adjusted *p*-value < 0.05, are listed.

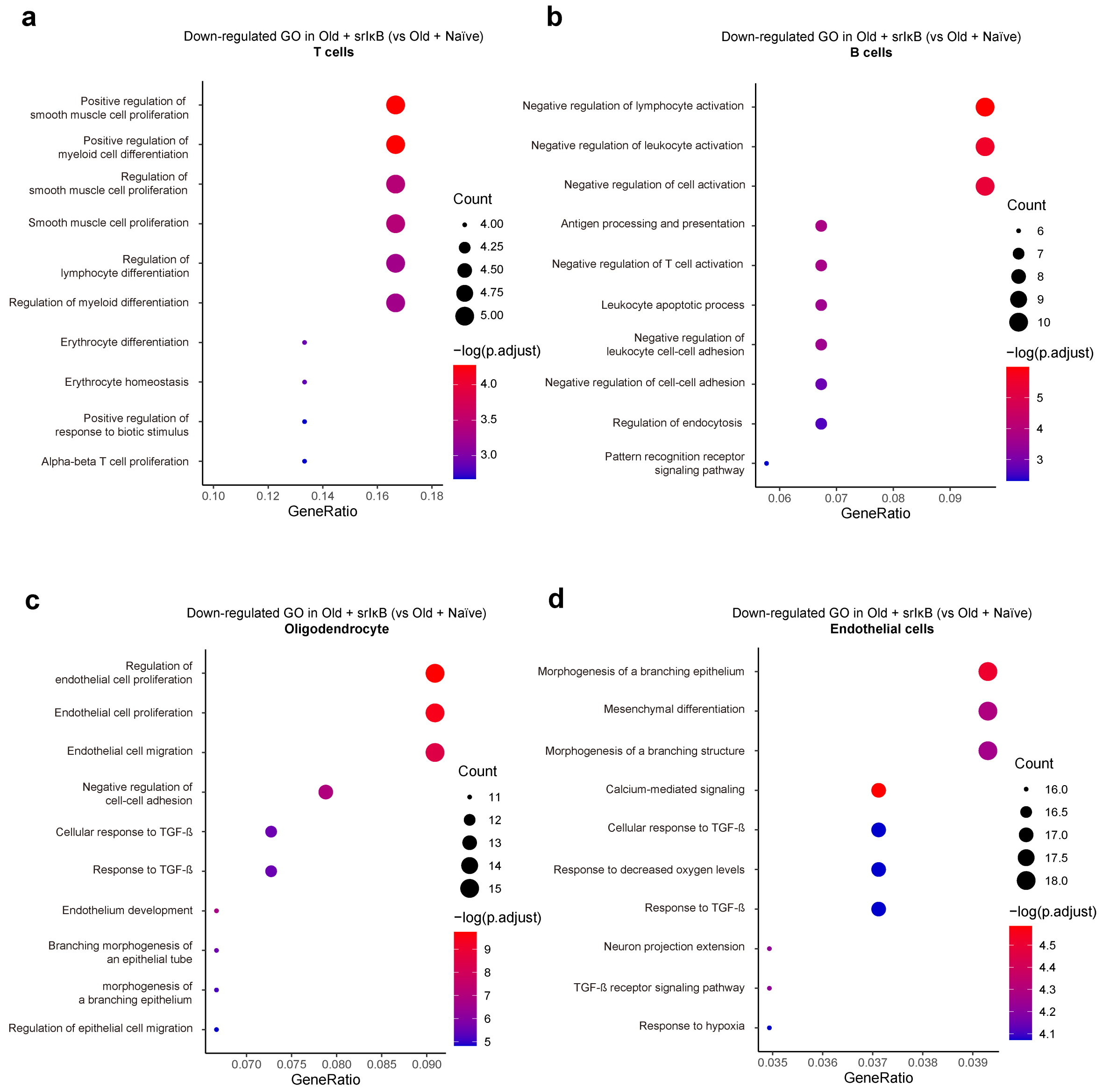

Supplementary Fig. 10. GO terms enriched among significantly downregulated DEGs. **a-d** GO terms enriched among significantly downregulated DEGs in T cells (**a**), B cells (**b**), oligodendrocyte (**c**), and endothelial cells (**d**) in Exo-srIκB-treated old mice compared with those in Exo-Naïve-treated old mice. The top 10 GO terms, according to the gene ratio, with BH-adjusted *p*-value < 0.05, are listed.

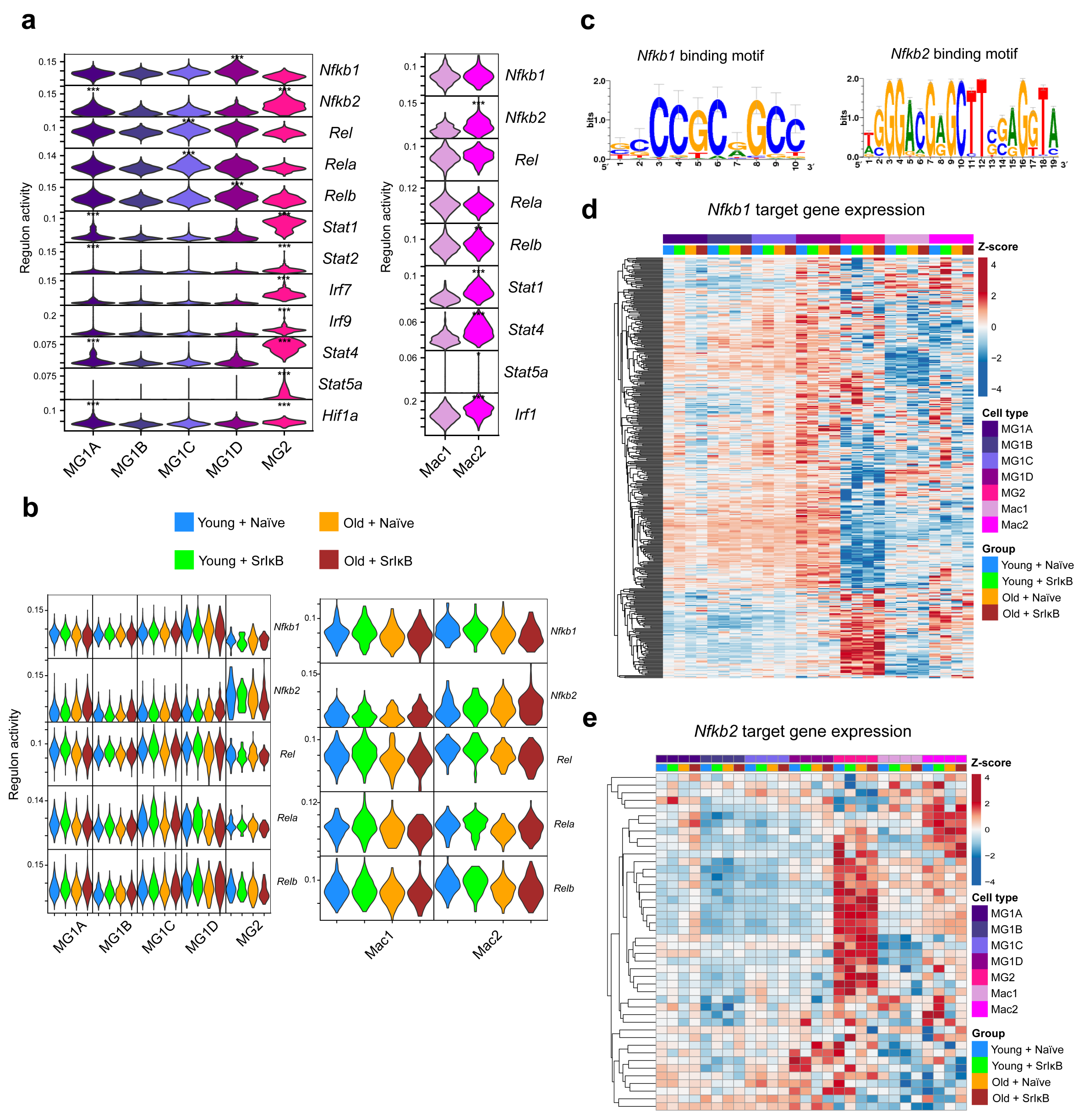

Supplementary Fig. 11. SCENIC analysis of microglia and macrophages.

a Activities of significantly upregulated regulons in one microglia or macrophage subtype compared with other microglia or macrophage subtypes. Wilcoxon rank sum tests with Bonferroni multiple comparison corrections were conducted to statistically determine cluster-enriched regulons. **p*-value < 0.05, ***p*-value < 0.01, ****p*-value < 0.001. **b** Comparison of NF-κB family regulon activities across the four groups within microglia and macrophages. **c** Sequence logos of *Nfkb1* and *Nfkb2* binding motifs. **d-e** Heatmap presenting the Z-scored,

normalized expression levels of *Nfkb1* (d) or *Nfkb2* (e) target genes across the four conditions within microglia and macrophages.

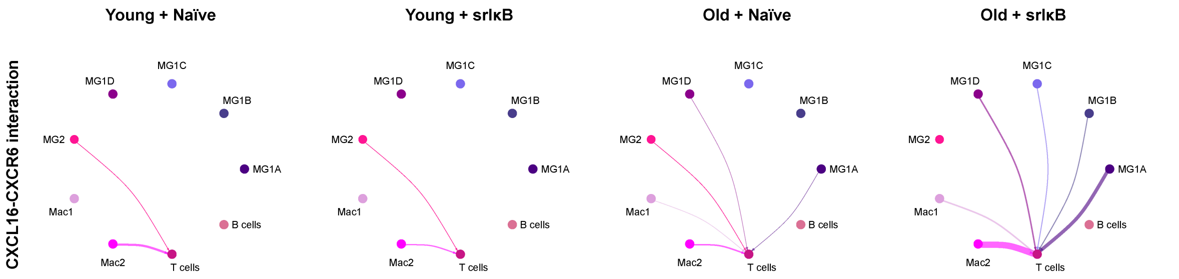

Supplementary Fig. 12. Circle plots depicting the interactions of CXCL16-CXCR6 from microglia and macrophages to T cells and B cells across four groups.

Edge colors represent the source cell types of the interaction, and edge widths are proportional to the interaction strength.

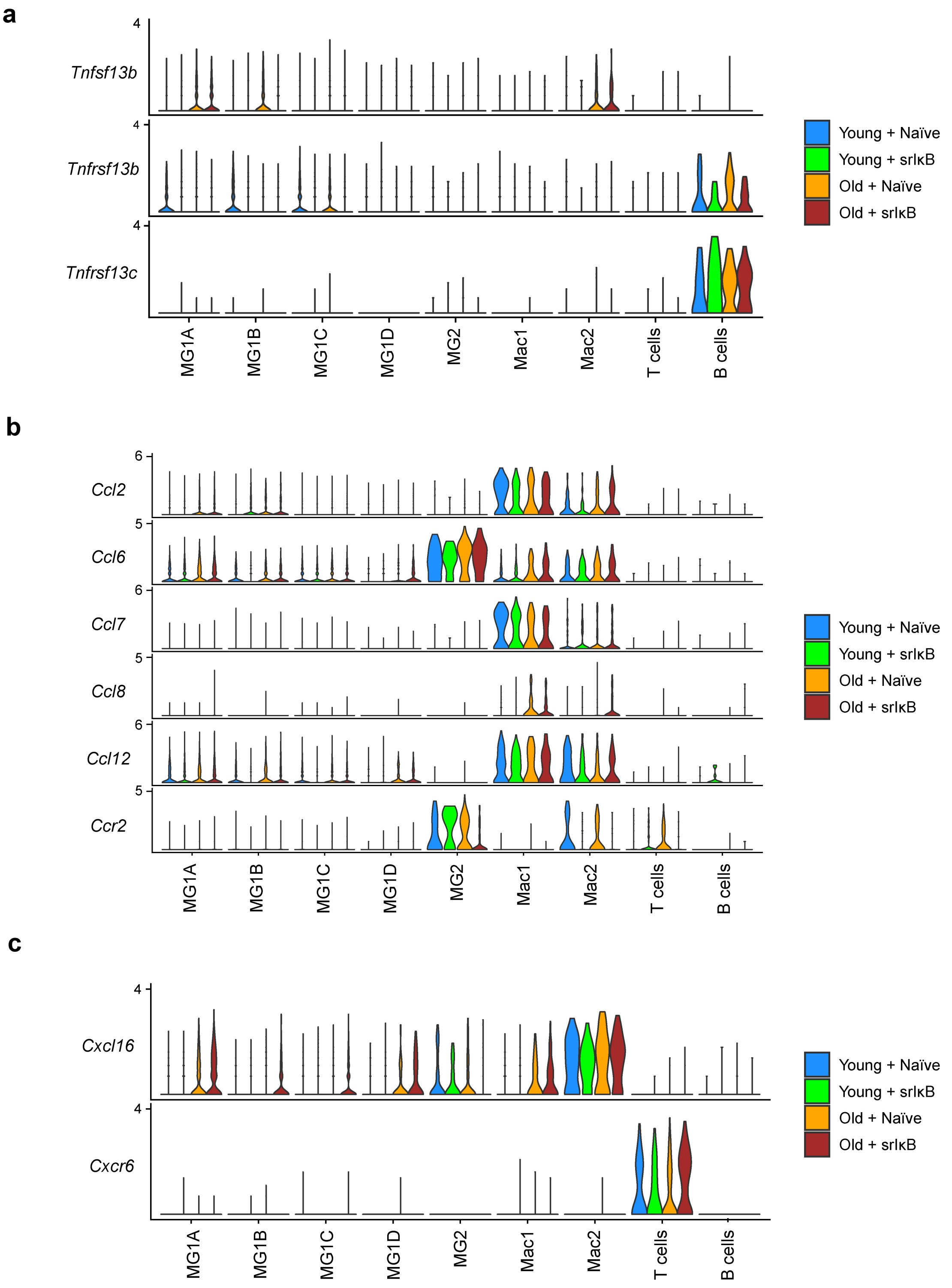

Supplementary Fig. 13. Violin plots of the normalized expression values for ligand-receptor pairs.

a-c Ligand–receptor pairs involved in BAFF signaling (**a**), CCR2-mediated signaling (**b**), and CXCL16-CXCR6 signaling (**c**) within all immune cells.

Supplementary Table. 1. List of genes used for calculating the demyelination-associated and myelination process-associated scores.

Demyelination_associated_scores	Myelination_process_associated_scores
Trf	Abca2
Apoe	Cd9
Gm42047	Degs1
Gm26917	Epb41l3
Cdkn1a	Mag
Neat1	Mal
Arap2	Mbp
Col5a3	Ndrg1
Syt4	Plp1
Serpina3n	Pmp22
Psap	Cntn2
Gdf15	Ugt8a
Abca1	Sirt2
Moxd1	Tspan2
H2-D1	Tppp
Ccng1	Ckap5
Hspa5	Arhgef10
Nupr1	Fa2h
Dock10	
Slc3a2	
Adamts1	
Slc7a1	
Slc38a2	
Sel1l	
Bcas1	
Ephx1	
Laptm4a	
Igfbp3	
Lrrc8c	
Gadd45b	
Srebf1	
mt-Co1	
Cdh6	
H3f3b	
Exoc4	

AI506816	
C1qa	
C1qb	
Ctsd	
Dock1	
Qpct	
Txnip	
Cebpg	
Tenm4	
C4b	
Epha5	
Gfap	
9330182L06Rik	
Mdm2	
Ctss	
Dusp10	
Igf1r	
Pvt1	
Frmd4a	
Itgb8	
Serpine2	
Gm2a	
Tma16	
Jun	
